# The microbiota knows: handling-stress and diet transform the microbial landscape in the gut content of rainbow trout in RAS

**DOI:** 10.1186/s42523-023-00253-9

**Published:** 2023-06-29

**Authors:** Marvin Suhr, Finn-Thorbjörn Fichtner-Grabowski, Henrike Seibel, Corinna Bang, Andre Franke, Carsten Schulz, Stéphanie Céline Hornburg

**Affiliations:** 1 Institute of Animal Nutrition and Physiology, Christian-Albrechts-University Kiel, Hermann-Rodewald-Straße 9, 24118 Kiel, Germany; 2 Fraunhofer Research Institution for Individualized and Cell-Based Medical Engineering, Hafentörn 3, 25761 Büsum, Germany; 3 Institute of Clinical Molecular Biology, Christian-Albrechts-University Kiel, University Hospital Schleswig-Holstein, Rosalind-Franklin-Str. 12, 24105 Kiel, Germany; 4 Institute of Animal Breeding and Husbandry, Christian-Albrechts-University Kiel, Hermann-Rodewald-Straße 6, 24118 Kiel, Germany

**Keywords:** Intestinal microbiome, Stress, Plant-based diets, Aquaculture, *Oncorhynchus mykiss*, 16S rRNA gene

## Abstract

**Background:**

The aim of the present study was to characterize the effects of handling stress on the microbiota in the intestinal gut contents of rainbow trout (*Oncorhynchus mykiss*) fed a plant-based diet from two different breeding lines (initial body weights: A: 124.69 g, B: 147.24 g). Diets were formulated in accordance with commercial trout diets differing in their respective protein sources: fishmeal (35% in fishmeal-based diet F, 7% in plant protein-based diet V) and plant-based proteins (47% in diet F, 73% in diet V). Experimental diets were provided for 59 days to all female trout in two separate recirculating aquaculture systems (RASs; mean temperature: A: 15.17 °C ± 0.44, B: 15.42 °C ± 0.38). Half of the fish in each RAS were chased with a fishing net twice per day to induce long-term stress (Group 1), while the other half were not exposed to stress (Group 0).

**Results:**

No differences in performance parameters were found between the treatment groups. By using 16S rRNA amplicon sequencing of the hypervariable region V3/V4, we examined the microbial community in the whole intestinal content of fish at the end of the trial. We discovered no significant differences in alpha diversity induced by diet or stress within either genetic trout line. However, the microbial composition was significantly driven by the interaction of stress and diet in trout line A. Otherwise, in trout line B, the main factor was stress. The communities of both breeding lines were predominantly colonized by bacteria from the phyla *Fusobacteriota, Firmicutes*, *Proteobacteria*, *Actinobacteriota*, and *Bacteroidota.* The most varying and abundant taxa were *Firmicutes* and *Fusobacteriota*, whereas at the genus level, *Cetobacterium* and *Mycoplasma* were key components in terms of adaptation. In trout line A, *Cetobacterium* abundance was affected by factor stress, and in trout line B, it was affected by the factor diet.

**Conclusion:**

We conclude that microbial gut composition, but neither microbial diversity nor fish performance, is highly influenced by stress handling, which also interacts with dietary protein sources. This influence varies between different genetic trout lines and depends on the fish’s life history.

**Supplementary Information:**

The online version contains supplementary material available at 10.1186/s42523-023-00253-9.

## Introduction

The global amount of fish produced in aquaculture has been constantly growing over the past 20 years, as is consumer demand [1]. This sector is a fundamental element in providing the human population with high-quality proteins. However, a profound demand for improved standards concerning animal welfare and nutrition, as well as sustainable production procedures, has been made by consumers. Despite aquaculture being a major and important pillar in food production, it lacks sustainability when it comes to feeding fish meal (FM) and fish oil made up of marine fish stocks, which have suffered from serious depletion in recent decades [1]. Therefore, it is necessary to use alternative protein sources with appropriate nutritious qualities in an aquafeed formulation. Many years of research have found suitable substitutes, such as oilseeds, legumes, cereal grains [2, 3], or insect meal [4]. Compelling quality measures for alternative feeds include low percentages of starch, fiber, and antinutrients [5].

Nevertheless, it is well established that dietary components change the environmental parameters for bacteria residing within the intestines of animals, the so-called microbiota [6, 7], which have earned an essential role in digestion, nutrient absorption, immune function, and protection against pathogens or modulation as part of the gut–brain axis [8]. Hence, diet formulation and the source of ingredients can alter the composition of the intestinal microbiota [7, 9] and thus the health condition of the host. Desai and colleagues revealed that a plant-based diet increased the overall richness, diversity, and abundance of lactic acid bacteria (LAB) from the phylum Firmicutes [10]. *Lactococcus* has been used as a probiotic on the basis of its inhibitory effects against fish pathogens shown in rainbow trout [11] and Atlantic salmon [12, 13]. It has also been reported that health beneficial commensals *Streptococcus*, *Leuconostoc*, and *Weissella* seem to be promoted when fish are fed a diet containing rapeseed oil and pea meal [14]. In contrast, Villasante and colleagues [15] revealed that a decreased abundance of LAB members *Leuconostoc* and *Weisella* while feeding a high carbohydrate diet is associated with fatty liver disease (hepatic steatosis) in Atlantic salmon. Additionally, juvenile rainbow trout are less susceptible to infection of *Yersinia ruckeri* and thus refer to a prebiotic effect of plant-derived products [14]. The effects of plant-based fish feed on the intestinal microbiome and thus host health are diverse and need to be evaluated when it comes to fish welfare in a stressful environment.

Moreover, lipase- and protease-producing bacteria are associated with a carnivorous diet, but the overall diversity compared to herbivorous fish decreases [16]. However, a constant set of bacteria seems to sustain and form a core microbiota in several species [17–20] *inter alia* rainbow trout [3, 21–24]. As several studies support, the core microbiota of rainbow trout is dominated by phyla *Bacteroidetes*, *Proteobacteria*, *Firmicutes*, and *Actinobacteria*, which seems to be consistent in wild-caught and selectively bred fish. Eventually, a well-balanced microbiome inhabits beneficial members that provide the host with valuable extracellular enzymes (carbohydrates, cellulases, phosphatases, esterases, lipases, and proteases), fatty acids, vitamins, and amino acids [25].

Besides an optimal nutrient supply, rearing conditions preferably free of handling, and group or environmental stress are desired for effective aquaculture. Handling stress, transportation, sorting, grading, and rearing conditions, such as temperature [26, 27] and stocking rates [3, 28], are common stress factors. Possible consequences include an increased susceptibility to pathogenic diseases [29]. Previous studies on zebrafish have pointed to a connection between stress resistance and the microbiome [30], and studies on rodents have revealed a severe change in the gut microbiome induced by stress [31, 32]. Further, in mammals, a negative impact on immunity and microbiome disruption induced by stress was reported, which may cause neurological disease [8], and in wild and cultured fish, stress is a trigger for increasing susceptibility to disease. Studies have been conducted on the negative impact of stress on the microbiome mediated via the stress hormone cortisol, resulting in individual changes in taxa [33]. An important role of the intestinal microbiome is to be a regulator of the gut–brain axis in vertebrates, which acts in the sympathetic and parasympathetic as well as the central nerval system. Considering that microbial colonization is influenced by diet type, it can be hypothesized that the stress response is also affected. Conversely, as mentioned previously, stress may disrupt microbial gut composition; thus, an interaction between these two factors must be considered. Based on the observed positive effects of bacteria promoted by plant-based diets, it can be hypothesized that fish fed plant-based diets might also be less susceptible to stress.

Several microbiome studies on productive livestock have revealed a strain-specific composition of the gut microbiome. Interestingly, distinct rainbow trout genetic lines have a specific composite microbiota that differs from other genetic lines [21, 28]. It has also been revealed that the response to a plant-based diet varies among the three isogenic trout lines based on transcriptomic profiling, where no distinction between genetic trout lines can be observed when fed a conventional FM diet [34]. Thus, it is likely that trout line-specific microbiota may be involved in the response to plant-based diets. Genetic selection for certain trout lines and their phenotypes in aquaculture might have an equal impact on the microbial community as environmental parameters and must be considered when analyzing the microbiome.

This study aimed to examine the following hypotheses: (i) the intestinal microbiome of rainbow trout is affected by an external handling stressor and (ii) there is an interaction effect between the inclusion of plant-based protein sources in the diet and handling stress on the microbial community. To evaluate the possible genetic influences on this interaction, two rainbow trout breeding lines were examined.

## Results

### Rainbow trout performance

At the end of the trial (day 59), the individual final body weights (FBWs) of 10 fish per tank (n = 240) did not differ significantly between the treatments. Further discussion of performance parameters, also in relation to molecular stress markers, has been published elsewhere [35]. Nevertheless, in order to discuss effects in relation to the intestinal microbiota comprehensively, individual FBW and initial body weight (IBW), as well as performance parameters based on group weights from day 50, are presented in Table 1: neither stress nor diet had a significant influence on performance parameters, except for a significantly increased daily feed intake (DFI) in trout from both breeding lines fed with diet V in comparison to fish fed with diet F (A: p < 0.001; B: p = 0.009). Since not all of the initially stocked fish were used for microbiota analysis, the number of samples of IBW and FBW varied.

**Table 1:** Initial (mean ± SD, n_A_ = 216, n_B_ = 192) and final body weights of individual rainbow trout (mean ± SD, n_A_ = 120, n_B_ = 120) and performance parameters (SGR, FCR, DFI, PER, and PRE) based on group weights from day 50 in relation to experimental groups (mean ± SD, n = 3)

Line	Treatment	IBW	FBW	SGR	FCR	DFI	PER	PRE
**A**	**0-V**	124.91 ± 0.23	347.80 ± 38.25	1.76 ± 0.03	0.88 ± 0.01	1.54 ± 0.00^B^	2.73 ± 0.04	44.32 ± 0.92
	**1-V**	124.71 ± 0.25	343.01 ± 45.00	1.73 ± 0.06	0.89 ± 0.03	1.54 ± 0.01^B^	2.69 ± 0.10	43.99 ± 5.98
	**0-F**	124.52 ± 0.66	359.82 ± 34.10	1.78 ± 0.02	0.85 ± 0.01	1.51 ± 0.01^A^	2.77 ± 0.02	45.79 ± 0.10
	**1-F**	124.62 ± 0.22	350.09 ± 40.50	1.72 ± 0.03	0.87 ± 0.01	1.49 ± 0.01^A^	2.70 ± 0.03	41.49 ± 3.48
**B**	**0-V**	146.90 ± 0.41	433.00 ± 58.23	1.74 ± 0.04	0.89 ± 0.02	1.54 ± 0.03^B^	2.71 ± 0.07	41.19 ± 0.42
	**1-V**	147.75 ± 0.76	421.73 ± 69.35	1.76 ± 0.07	0.87 ± 0.03	1.53 ± 0.01^B^	2.76 ± 0.10	39.40 ± 6.48
	**0-F**	147.15 ± 0.41	410.63 ± 43.93	1.75 ± 0.05	0.85 ± 0.02	1.49 ± 0.01^A^	2.75 ± 0.07	45.48 ± 0.90
	**1-F**	147.15 ± 0.51	415.29 ± 56.39	1.78 ± 0.02	0.85 ± 0.01	1.51 ± 0.00^A^	2.77 ± 0.04	44.55 ± 0.91

*IBW* initial body weight (g), *FBW* final body weight (g), *SGR* specific growth rate, *FCR* feed conversion ratio, *DFI* daily feed intake, *PER* protein efficiency ratio, *PRE* protein retention efficiency. For the formula, see the supplementary file. Values in the same column with different superscript letters within each genetic trout line are significantly different (p < 0.05). Unstressed group (0), stressed group (1), fishmeal diet (F), plant-based diet (V).

### 16S rRNA bacterial assignment from the intestinal gut content of rainbow trout

To assess the intestinal microbial composition of individual trout, bacterial DNA extracted from total gut content at the 16S rRNA V3–V4 region was sequenced. Demultiplexed Illumina MiSeq reads comprised of 2 × 300 bp were imported in Qiime2, with a raw total read count of 7,458,158 per read direction. After primer trimming and denoising, 5,408,391 reads, which correspond to a total loss of 27.48%, were left for further analysis. After taxonomic classification, amplicon sequence variants (ASVs) identified as cyanobacteria or mitochondrial origins were removed from the dataset because they were considered contaminated. Samples from batches with contaminated negative controls (i.e., with reads originating from fish intestinal microbes) were removed from further analysis. Generally, samples were considered contaminated when the extraction control from that run had a considerably high number of reads. In total, 145 samples were left for further analysis, comprising 41 initial samples and 104 treatment samples. For the experimental dataset (without RAS samples), a total ASV count of 764 (69 samples) for Group A and 540 (35 samples) for Group B were left for further downstream analysis. Finally, 2,446,407 reads remained for downstream analysis, including initial sampling (A: n = 21, B: n = 20) and experimental samples. The average number of reads was 16,872 (without rarefaction).

**Fig. 1:**
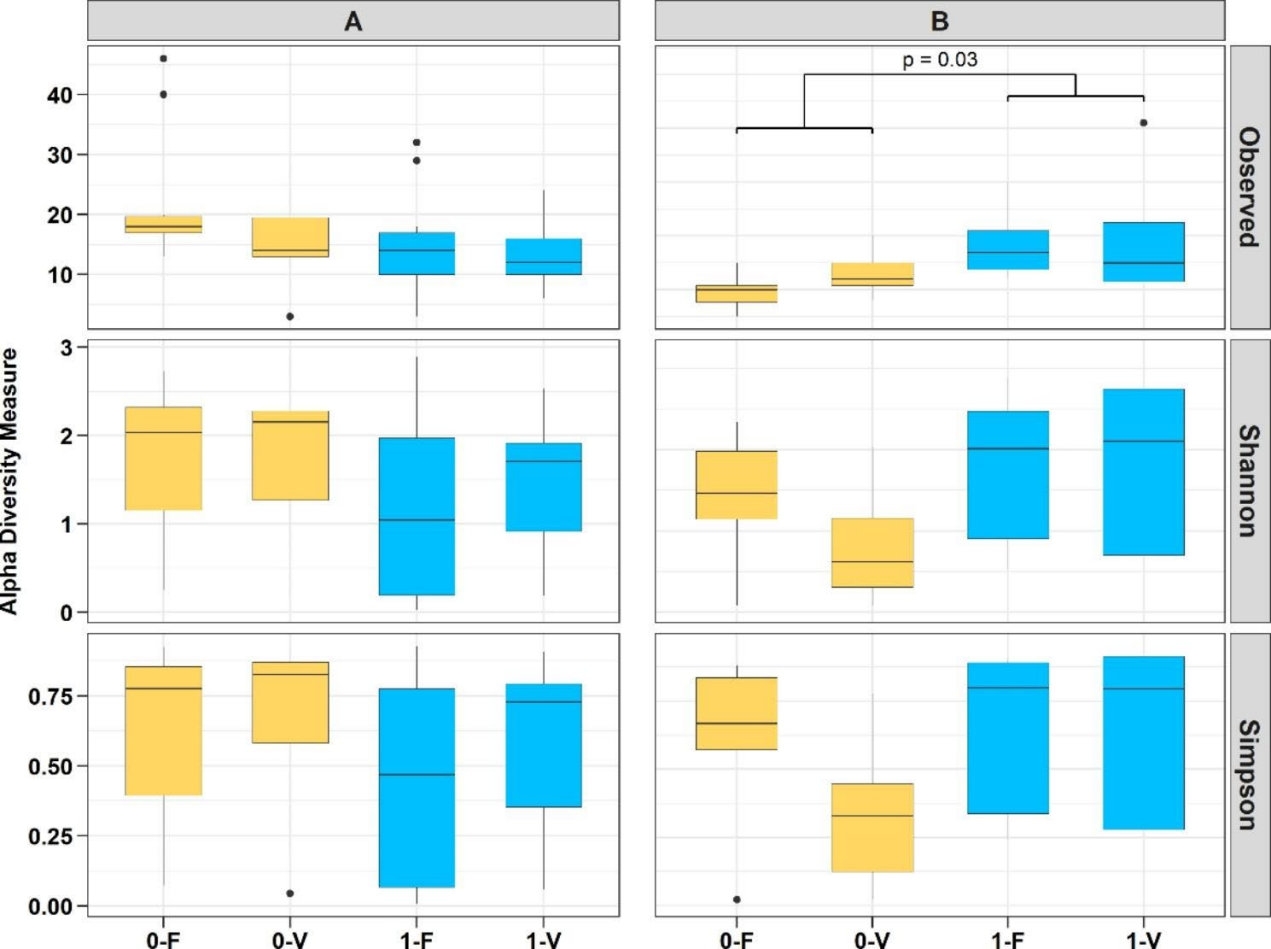
Alpha diversity indices including observed ASVs, Shannon diversity, and Simpson indexes of the four treatments from rainbow trout gut content after 59 days of feeding. The left panel shows trout line A (0-F, 0-V, 1-F, 1-V), and the right panel shows trout line B (0-F, 0-V, 1-F, 1-V). Boxes are colored according to factor stress, where the unstressed group (0) is yellow and the stressed group (1) is blue. FM-based diet (F) and plant-based diet (V). n = 3

### The impact of stress on microbial diversity

Alpha diversity measures, including observed ASVs (richness) as a qualitative parameter and Shannon diversity index and Simpson index as quantitative parameters, were estimated based on genus-level data to compare taxonomic diversity between treatments. The average observed ASVs across all treatments within each genetic trout line did not vary significantly between A (~ 28) and B (~ 29). Evaluation of trout line A revealed no significant interaction or any significant effect of diet or stress on any of the diversity measures. Considering richness and evenness (Shannon), diversity in treatment groups 0-F and 0-V was higher on average than in treatment groups 1-F and 1-V. However, in trout line B, we found significantly higher observed ASVs for stressed fish in contrast to unstressed fish, independent of dietary composition (p = 0.03). This effect was also observed for the Shannon and Simpson indexes, although it was not significant.

### Beta-diversity is affected by stress and diet

**Fig. 2:**
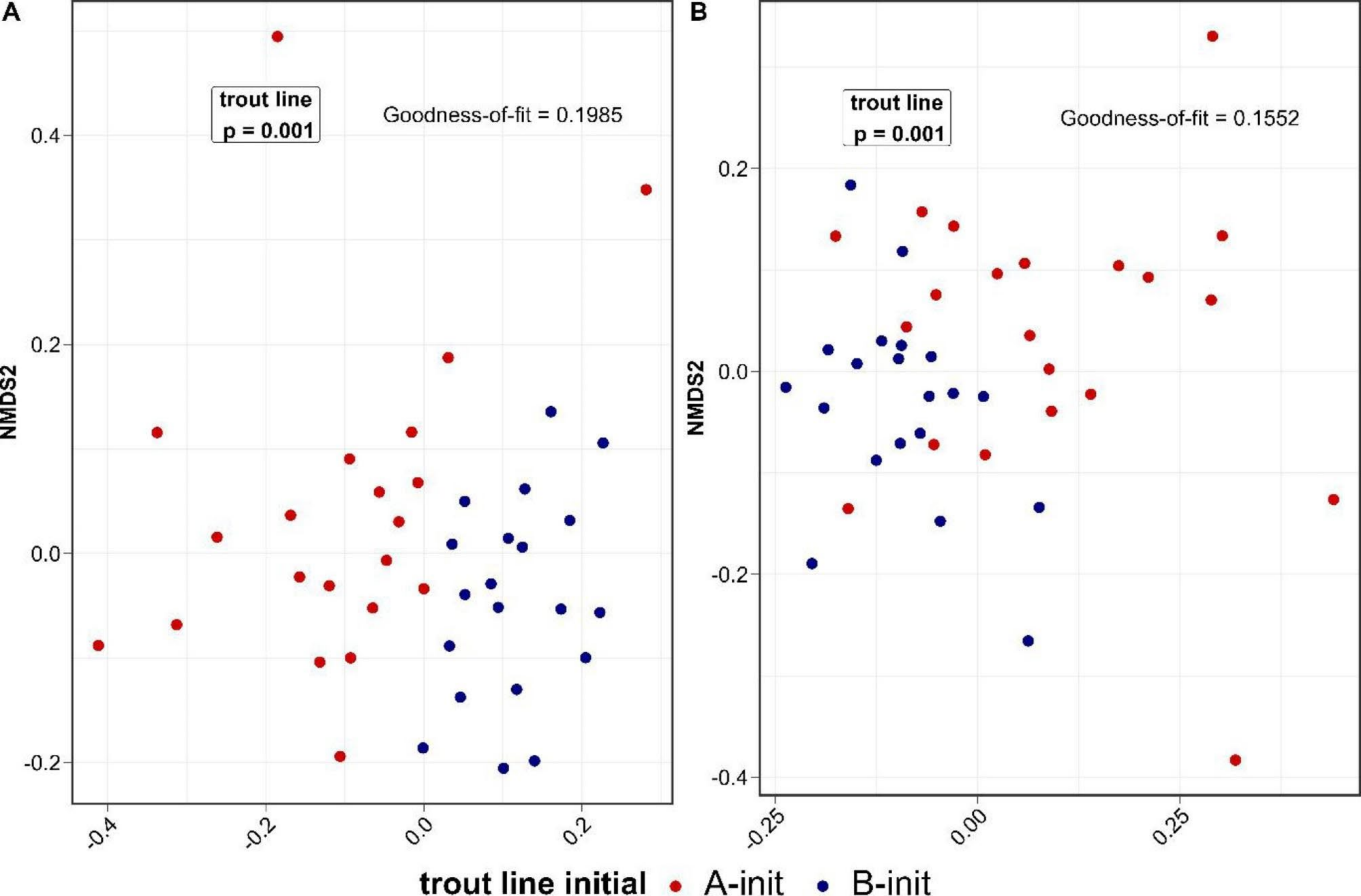
Microbial composition comparison of rainbow trout gut contents among initial samples of both genetic trout lines on genus-level data using NMDS on unweighted **(A)** and weighted UniFrac **(B)** distances. Initial samples are colored in dark red (A-init) and dark blue (B-init)

First, we intended to determine the overall microbial community composition altered by diet-by-stress treatments in genetic trout lines. For nonmetric multidimensional scaling (NMDS), we calculated unweighted UniFrac distances to evaluate community structure by integrating phylogenetic information and presence or absence information and further weighted UniFrac distances, which additionally included abundance data based on genus-level data. First, a model that included only initial samples from A and B demonstrated significant differences between both genetic trout lines prior to the trial (Fig. 2A, unweighted, p = 0.001; Fig. 2B: weighted, p = 0.001; permutational multivariate analysis of variance [PERMANOVA], Supplementary Table T3).

**Fig. 3:**
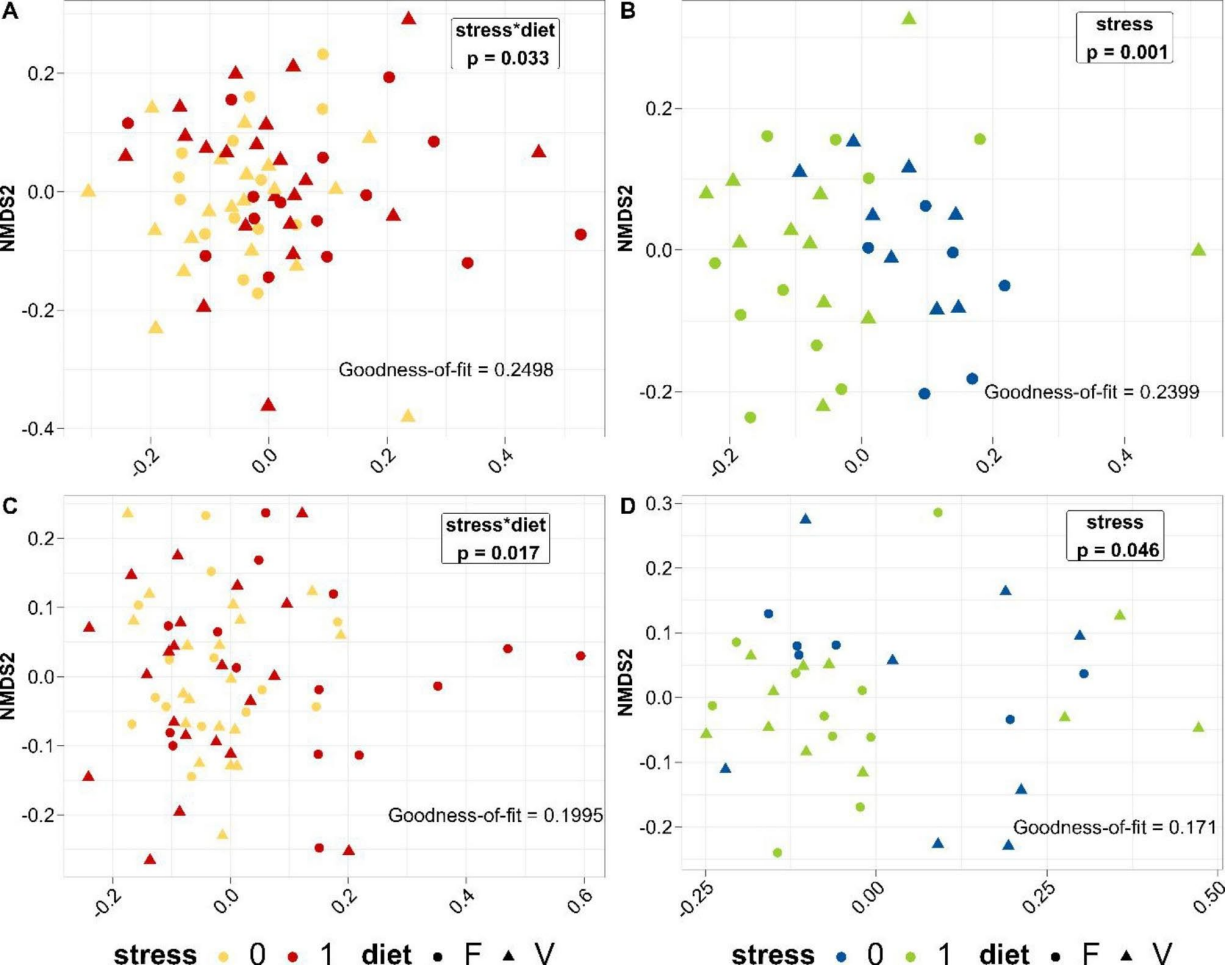
Microbial composition comparison among treatments on genus level data from rainbow trout gut content. NMDS on unweighted UniFrac distances for trout lines A and B (A, B) and weighted UniFrac distances for trout lines A and B (C, D). The stress factor is colored gold (0) and red (1) for trout line A and green (0) and blue (1) for trout line B. Diets are depicted with circles for the FM diet (F) and triangles for the plant-based diet (V)

Graphical representation of unweighted UniFrac of trout line A (Fig. 3A) revealed no clustering by treatment but rather whether the fish were stressed or not. However, the PERMANOVA results revealed significant differences in microbial composition among treatments, explained by the interaction factor of stress and diet (p = 0.033). This was also confirmed by weighted UniFrac (PERMANOVA, p = 0.017, Fig. 3C). Results of pairwise statistical comparison in trout line A indicated scarce significant differences between treatment A-0-V and A-1-V (p = 0.055) in unweighted UniFrac but significant differences in stressed trout fed either diet F or V (p = 0.046) in weighted UniFrac. Additionally, differences were also observed in treatments fed diet F, where the stress factor had a significant impact (p = 0.028).

Individuals from line B significantly differed by factor stress (PERMANOVA, unweighted: p = 0.001, weighted: p = 0.046), which was also confirmed by NMDS visualization (Fig. 3B, unweighted; Fig. 3D, weighted). However, no interaction between diet and stress was observed in trout line B (PERMANOVA, unweighted: p = 0.19, weighted: p = 0.111). The variability of microbial communities within groups was further tested by dispersion analysis incorporating both UniFrac distances on genus-level data. Consistent with previous results, significantly increased values for dispersion (distance to centroid for each sample in the associated group) in trout line B for stressed fish across the diet were detected in the unweighted UniFrac distance (analysis of variance [ANOVA], p = 0.041, Supplementary Table T3). No significant differences in trout line A were observed.

### Taxonomical composition of microbial communities

**Fig. 4:**
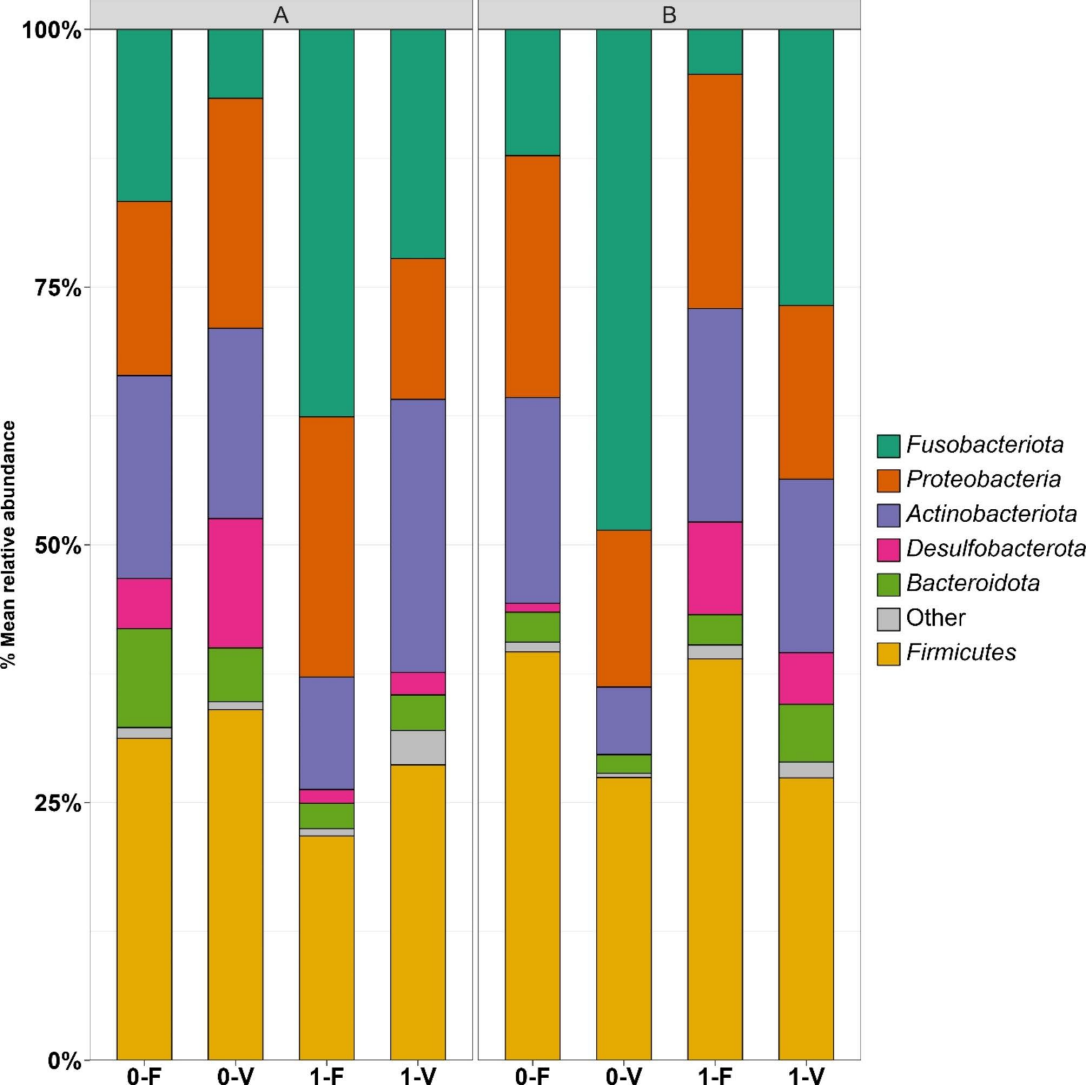
Mean relative abundance (%) at the phylum level of the gut content microbiota from rainbow trout line A in the left and B in the right panel. Each bar represents a diet-stress combination treatment within the genetic trout lines. Phyla with a representation of relative abundance < 0.05 were pooled in the category ‘Other’. The arrangement of the bars is based on abundance, except for the most abundant phyla, which is placed at the bottom for legibility. The mean value joins the data from each individual fish for the corresponding treatment across the three tanks. Unstressed group (0), stressed group (1), fishmeal diet (F), plant-based diet (V)

To further analyze the microbial gut composition of rainbow trout, we examined the relative abundances on the phylum and genus levels. Over all analyzed libraries, 25 phyla were observed, from which the top six dominant phyla across all treatments in trout lines A and B were *Fusobacteriota, Firmicutes*, *Proteobacteria*, *Actinobacteriota*, *Desulfobacterota*, and *Bacteroidota*, as shown in Fig. 4. Phyla with a relative abundance < 0.05 were declared as ‘Other.’ Multiple contrast tests between treatments revealed that intestinal microbiota from trout line A exposed to stress in both diets was significantly increased with *Fusobacteriota* (p = 0.04) compared to trout with no stress exposure. When comparing *Fusobacteriota* abundance within stress groups, abundance decreased when diet V was fed (not significant). Contrary to trout line A, the increased abundance of *Fusobacteriota* in both stress groups in trout line B was associated with diet V (0: p = 0.045; 1: not significant). In addition, trout not exposed to stress had a higher abundance of *Fusobacteriota* within their respective diet group.

The abundance of *Firmicutes* is > 25% in all treatments, except for A-1-F (22%), which did not deviate significantly from the mean abundance. *Firmicutes* represents the most abundant phyla across all treatments. In trout line A, although not significant, *Firmicutes* abundance was reduced in trout exposed to stress in both diets compared to trout that were not stressed. A different effect in trout line B was observed, where diet V in the stressed and unstressed groups reduced the abundance of *Firmicutes. Proteobacteria* was represented by 14–25% and 15–23% in trout lines A and B, respectively. Bacteria from the phylum *Desulfobacterota* were highest in the unstressed group, disregarding the factor diet in trout line A, and an inverse effect, where the highest abundance was in the stressed groups, was observed in trout line B. Less variation was observed in *Actinobacteriota* and *Bacteroidota*.

Initial sampling from breeding line A (A-init) was composed of a high abundance of Desulfobacterota (33%) and *Fusobacteriota* (21%) and a low abundance of *Verrucomirobiota* (3%), which was present only in the initial samplings. Half of the microbiota in the initial sampling from trout line B was composed of *Firmicutes* (50%), followed by less abundant taxa *Proteobacteria* (19%) and *Actinobacteriota* (15%). Further bacterial taxa found in the initial sampling of both genetic trout lines can be found in the supplementary material section (Figure S3).

**Fig. 5:**
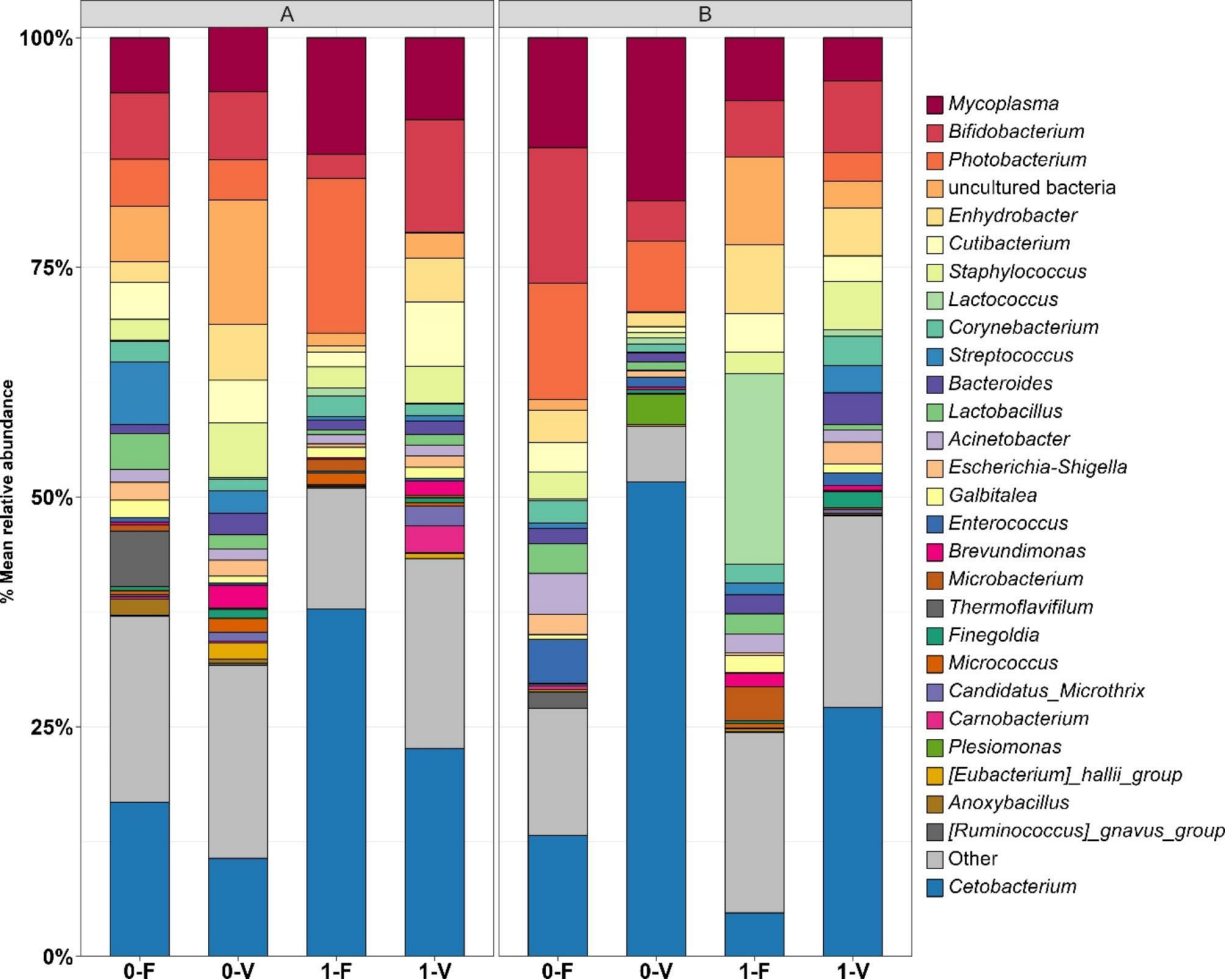
Mean relative abundance (%) at the genus level of the gut content microbiota from rainbow trout line A in the left and B in the right panel. Each bar represents a diet * stress combination treatment within the genetic trout lines. Genera, with a representation of 1.5%, were pooled in the category ‘Other’. The arrangement of the bars is based on abundance, except for the most abundant phyla, which is placed at the bottom for legibility. The mean value joins the data for each individual fish for the corresponding treatment across the three tanks. Unstressed group (0), stressed group (1), fishmeal diet (F), plant-based diet (V)

Classification down to the genus level was achieved for 265 and 211 ASVs in lines A and B, respectively. Taxa with an abundance of less than 0.01% were declared as ‘Other,’ which led to 37 genera, as displayed in Fig. 5. The phylum *Fusobacteriota* comprised a single genus in both genetic trout lines, *Cetobacterium*, and was thus close to absence in B-init (Supplementary Figure S4). Thus, abundances from this phylum resembles the genus distribution. Stressed fish from trout line A showed significantly increased abundances in *Bifidobacterium* (12%, p = 0.023) and *Enhydrobacter* (4%, p = 0.036) when fed diet V. Otherwise, an increased abundance of *Staphylococcus* (6%, p = 0.036) was observed in non-stressed fish when fed diet V in contrast to diet F. In treatment A-1-F, significantly reduced abundance of *Lactobacillus* (0.5%, p = 0.011) compared to A-0-F (4%) was observed. Furthermore, diet V significantly increased the appearance of *Brevundimonas* (2%, p = 0.029) and *Candidatus Microhrix* (2%, p = 0.023) when fish were stressed in contrast to diet F when stressed.

In trout line B, the highest abundance of *Lactococcus* (21%) and *Plesiomonas* (3%) was observed in treatment B-0-V compared to all other treatments. The highest abundance of *Mycoplasma* was found in unstressed fish fed diet V (19%). Although not significant, *Mycoplasma* was lower in abundance (mean: 6%), and *Streptococcus was* higher in abundance (mean: 2%) in fish that were stressed despite the factor diet. *Carnobacterium* was not present in any treatments of trout line B, except for B-1-F (0.5%). *Bifidobacterium* had the highest abundance (15%) in treatment 0-F, whereas in the other treatments, no particular variation was observed.

To determine which microbes contributed the most to the observed differences in the microbial composition of intestinal gut contents, a linear discriminant analysis of effect size (LefSe) was conducted. Several taxa were identified as significantly differential abundant among the treatments based on the estimated effect size. From these results, Table 2 shows the corresponding treatments and linear discriminant analysis (LDA) scores for each significant genus. A prominent phylotype, *Cetobacterium* from the phylum *Fusobacteriota*, was significantly enriched in the very distinct treatment groups A-1-F and B-0-V. Most of the reads were classified as *uncultured bacterium*, while some could be assigned as *Cetobacterium somerae*. Furthermore, *Mycoplasma* (B-0-V), *Photobacterium* (A-1-F), *Plesiomonas* (B-0-V), *Acinetobacter* (B-0-F), *Bifidobacterium* (A-1-V), and *Lactobacillus* (A-0-F) were significantly enriched in the respective treatments.

**Table 2:** Linear discriminant analysis effect size (LEfSe) results of relevant features significantly (p < 0.05) characterizing experimental treatments

Treatment	Phylum	Genus	ef_lda	padj
A-0-F	*Firmicutes*	*Lactobacillus*	4.358	0.002
A-0-V	*Bacteroidota*	*Bacteroides*	4.173	0.035
A-0-V	*Proteobacteria*	*Rhodobacteraceae_uncultured*	3.400	0.018
A-1-F	*Fusobacteriota*	*Cetobacterium*	4.892	0.002
A-1-F	*Proteobacteria*	*Photobacterium*	4.573	0.002
A-1-F	*Proteobacteria*	*Plesiomonas*	3.957	0.015
A-1-F	*Actinobacteriota*	*Dietzia*	3.758	0.004
A-1-V	*Actinobacteriota*	*Bifidobacterium*	4.415	0.020
A-1-V	*Actinobacteriota*	*Candidatus_Microthrix*	4.070	0.035
B-0-F	*Proteobacteria*	*Acinetobacter*	4.431	0.033
B-0-F	*Proteobacteria*	*Schlegelella*	3.893	0.040
B-0-F	*Proteobacteria*	*Roseomonas*	3.616	0.033
B-0-V	*Fusobacteriota*	*Cetobacterium*	4.879	0.008
B-0-V	*Firmicutes*	*Mycoplasma*	4.725	0.041
B-0-V	*Proteobacteria*	*Plesiomonas*	4.445	0.004
B-0-V	*Actinobacteriota*	*Rhodococcus*	3.720	0.029
B-1-F	*Actinobacteriota*	*Microbacteriaceae*	4.265	0.036
B-1-F	*Verrucomicrobiota*	*Luteolibacter*	3.827	0.036
B-1-F	*Proteobacteria*	*Bosea*	3.717	0.047
B-1-F	*Proteobacteria*	*Rhizobiaceae*	3.695	0.029
B-1-F	*Actinobacteriota*	*Rothia*	3.480	0.029

## Discussion

The present study is the first to investigate the microbial community composition and performance of two rainbow trout (*Oncorhynchus mykiss*) genetic lines in RAS exposed to handling stress while fed plant-based diets—important parameters in fish farming with regard to animal health and welfare. We believe that this data sheds more light in the commensal relationship of host and bacteria, with a focus on allochthonous (transient) inhabitants. Recently, attention has been paid to the intestinal microbiome of aquaculture species, as it plays a key role in health, growth, and disease status [36, 37]. Consequently, investigating the interaction of factors that shape the microbiome, such as diet, stress, environment, or genetic state, among others, is crucial. By conducting marker gene sequencing at the hypervariable V3/V4 region of the 16S rRNA gene of microbes residing in the intestinal gut material of rainbow trout haltered in RAS, modifications of the microbial landscape in the gut content of rainbow trout through interacting effects of handling stress and plant-based diets were evaluated. In this study, as a FM replacement (diet V), we used 20.5% soy protein concentrate, 5% soybean meal, and 15.2% wheat gluten. Otherwise, diet F comprised 6% soy bean concentrate, no soybean meal at all, 6% wheat gluten, and 35% fishmeal as protein sources. To simulate handling stress and thus trying to obtain its impact on microbial communities in combination with diet, fish were exposed to chasing with a net as an external stress stimulus.

### Fish performance

The experimental diets used in this trial were formulated according to commercial trout diets with varying inclusion levels of plant-based proteins and met all nutritive requirements for rainbow trout. Therefore, the single effects of the experimental diets on fish performance were not expected and were not observed. However, we hypothesized effects on performance parameters through the applied handling stressor and through an interaction of both factors (diet*stress), as we expected the microbiome to be a physiological modulator of the stress response in fish [30]. Surprisingly, we also did not observe single effects of stress or effects of a diet–stress interaction in both breeding lines on fish performance, except for a significantly increased DFI associated with diet V. Daily feed intake varies with 0.03 g*d^− 1^ on average between the two dietary treatment groups, which can be important in large-scale production but is supposedly of minor biological relevance when examining intestinal microbiota.

### Alpha diversity

Mean microbial richness and diversity parameters did not significantly differ between treatment groups, except for a gain in observed ASVs in stressed fish from trout line B, independent of the experimental diet fed. Indeed, samples of treatment groups B-0-F and B-0-V have much fewer sequencing reads after filtering and decontamination procedures since less than half of the samples are left in comparison to all other treatments. This might explain the statistical differences found in the observed ASVs. However, the observed values in this study are still in the range of what has been observed in other microbiota studies in rainbow trout [3, 28], even though there are several studies that report less [38] or even an increased number of taxa [22, 23, 39] in the intestinal contents of rainbow trout, which might be a consequence of missing standardization in metagenomic profiling studies of bacteria since, for example, several hypervariable regions and sampling procedures can be found in the literature regarding the same species. Nevertheless, the significant effect of handling stress on observed ASVs in trout line B should not be neglected. The increase in bacterial taxa could reflect a protective mechanism of the intestine against external stress.

### Community composition of intestinal gut microbiota

Analysis of the intestinal gut content at the phylum level revealed that five taxa, namely *Fusobacteriota, Firmicutes*, *Proteobacteria*, *Actinobacteriota*, and *Bacteroidota*, dominated the communities in both breeding lines and across all treatments. Our findings agree with previous studies that defined these phyla as major taxonomic groups of the vertebrate gastrointestinal tract [40] or yet more important in studies on rainbow trout [3, 14, 23, 41] or fish in general [42]. Exceptional is the presence of *Desulfobacterota*, which is not described in the previously mentioned ‘core-microbiota’ in fish. Previously classified as *Deltaproteobacteria*, this anaerobic group represents sulfate-reducing and moreover fermentative syntrophic bacteria [43]. The highest abundance was detected in A-init, and evaluating other treatments, this phyla can be classified as a low abundant phylum similar as in [41]. In agreement with a previous study, the domination of *Fusobacteriota* and *Firmicutes* in the intestinal gut microbiota of rainbow trout [44] were *Cetobacterium* and *Mycoplasma*, which represent the predominant genera in the bacterial community.

There seems to be no comprehensive interaction of diet and stress modulating the frequency of a particular phylum in either genetic trout lines or rainbow trout, respectively, when evaluating the statistical results of the relative abundance data (Supplementary Table T5, T6). Although bacteria from the phylum *Firmicutes* displayed the dominant phyla in all treatments in both breeding lines, no significant statistical association with a certain diet or stress situation was found. However, several studies [10, 14, 45] revealed that plant-based proteins in the diet favor the presence of *Firmicutes*, and another study showed that high-temperature stress is able to decrease the abundance of bacteria belonging to that phyla [27]. Although not significant, a trend of increased *Firmicutes* occurrence could be observed in the primarily FM-based diet in trout line B, which is opposed to the previously mentioned association with a plant-based diet. Another study analyzing the stress stimuli hypoxia and high fish density on the microbiome of the skin mucus layer in brook charrs found a significant increase in pathogenic microbes and a decrease in probiotic-like bacteria [46]. In the present study, no such primary regulation of certain bacterial clades could be assigned to the applied parameters, possibly due to the applicable inter-sample variation within the treatments. A further conclusion of the previously mentioned studies is the increased abundance of *Proteobacteriota* in fish fed an FM-based diet, while diets of plant origin seem to promote bacteria from the phylum *Firmicutes*. In trout from line B, though not significant, a similar effect can be observed. However, bacteria from the phylum *Fusobacteriot*a must be considered within the ratio *Firmicutes:Proteobacteria*. The phyla *Actinobacteriota, Desulfobacterota*, and *Bacteroidota* show only minor changes in abundance and no significant association with diet or stress.

### Prevalence of specific genera in certain treatments

Our analysis revealed that the second most abundant phyla was *Fusobacteriota*. Interestingly, 99.97% of all ASVs belong to the genus *Cetobacterium*. Similar observations were made in omnivorous fish, such as common carp [47], with 93.94% coverage of *Fusobacteriota* and 8,081 sequences out of 8,085 of *Fusobacteriota* in a study by van Kessel et al. [48]. Furthermore, a diet-related study of largemouth bass revealed up to 89.9% coverge by *Cetobacterium* [49, 50]. In humans, *Cetobacterium* is associated with protein and carbohydrate fermentation [51], while in freshwater fish, *Cetobacterium* is known for vitamin B12 and short-chain fatty acids [52]. These attributes correspond to the results of the aforementioned studies in that *Cetobacterium* abundance seems to be associated with an omnivorous–herbivorous diet in freshwater fish [52, 53]. The presence of *Cetobacterium* in the gastrointestinal tract of rainbow trout was found to be the most abundant genus in the mucus layer of rainbow trout [54, 55], and yet another study suggests an association with carnivorous fish [16]. While this study revealed a strong association with two certain treatments, the marked presence of *Cetobacterium* was found in all gut materials of the sampled rainbow trout. Such a general increased abundance of that species leads to the assumption that *Cetobacterium* plays a key role in the digestion of certain nutrients and might be crucial for vitamin B12 metabolism in rainbow trout. Further studies in this context need to be conducted to address the function of *Cetobacterium* within this microenvironment.

Another recurring bacterial genus is *Mycoplasma—*in this data, allocated to the phylum *Firmicutes*, formerly classified as *Tenericutes* [56]. It has been reported that *Mycoplasma* portrays a dominant representative commensal in the gastrointestinal tract regardless of compartmentation of rainbow trout [27, 28, 41, 55, 57–59] and other salmonids [60–64] while found to be pathogenic in human hosts [65]. A few studies have observed *Mycoplasma* to be the most abundant taxa in rainbow trout gut digesta, and the quantity was unaffected by the respective diet [66, 67]. In comparison to sequences obtained from gut digesta, *Mycoplasma* has been found to be more associated with the mucus layer within the alimentary tract [68]. Furthermore, *Mycoplasma* abundance has been found to be positively correlated with FBW and disease resilience in Atlantic salmon [62, 69]. Our findings support the hypothesis that *Mycoplasma* is a dominant microbe in the gut contents of salmonid hosts. While still lacking an exact detailed assertion of the relationship and function between microbe and host, a long and close evolutionary and ecological commensal connection has been proposed. As salmonids are ammonotelic and are not able to *de novo* synthesize arginine (and its derivates), one explanation for the strong presence of *Mycoplasma* in general may be its ability to detoxicate ammonia via its pathway by building up a nitrogen carrier. While in the aforementioned studies the focus was on metagenomic analysis of *Mycoplasma*, transcriptomic data of such scenarios may unravel the functional relationship between this microbe and the host. Because it is frequently reported in rainbow trout, the omnipresence of *Mycoplasma* in gut contents is unequivocal, regardless of what environmental conditions or diets are being applied. In particular, in the present treatment, no significant patterns between the conditions were determined. Although LEfSe results indicated substantive association of *Mycoplasma* with plant-based diet and no stress exposure in trout line B, a general connection between diet and stress cannot be related; thus, these findings underline the ubiquity of *Mycoplasma* in the alimentary tract of rainbow trout.

Bacteria from the genus *Bifidobacterium* were found to be present in all treatments, with a marked dominance in stressed fish fed a plant-based diet in genetic trout line A. Otherwise, in trout line B, such bacteria had their highest abundance in unstressed fish fed an FM-based diet. Representatives from the genus *Bifidobacterium* are heterofermentative microorganisms and play a major role in the utilization of carbohydrates in several organisms. Because of their beneficial properties, they have been used as probiotics in rainbow trout, where they are able to enhance growth performance and nutrient utilization [70]. While *Bifidobacterium* are common and major representatives of the mammalian gut, reports about their presence in rainbow trout and other finfish are scarce [71]. In one of our previous studies, we connected the abundance of bacteria belonging to the order of *Bifidobacteriales* to a plant-based diet in juvenile brown trout [9]. However, in the present study, abundance profiling of *Bifidobacterium* did not reveal a strong connection to any of the tested parameters. The overall (equal) presence of this taxa might be caused by the constant energy content across the diets.

### Stress as the main regulator of the microbiome

The genetic trout lines used in this study were raised in two distinct aquaculture breeding facilities and thus experienced high environmental variation. Because of the different life histories, conditions prior to the experiment, the different genotypes (genetic variation), and deviating adaptation parameters (time, feeding, etc.), we expected a predetermined bacterial community in each genetic trout line due to early colonization events prior to the experiment. This hypothesis was supported by our findings that the intestinal gut microbiota compositions from trout lines A and B prior to the experiment clearly separated with regard to phylogenetic beta diversity analysis. Which of the multiple factors or a combination of all are responsible for these observations is not evident, but such microbial imprinting has also been observed in rainbow trout [28] among other species [17, 72]. Therefore, we analyzed both breeding lines separately. Suggesting that gut microbiome modification is affected by both diet and stress in general, early colonization can be resolved and supports the general idea of a flexible and malleable microbiome at several life stages [7, 9, 14]. Interestingly, the husbandry conditions in the RAS were arranged to overcome the initial distinction by the early colonization of both genetic trout lines, suggesting an alignment of the microbial landscape due to the experimental setup at the end of the experiment. This indicates that life history effects on the microbial population can be modulated again. Nevertheless, there are differences between the two genetic trout lines in their response to the experimental treatments, suggesting a genetic impact on the stress–diet–microbiome interaction.

In trout line A, the microbiota was altered through the interaction of the factors of diet and stress. Within each respective diet, the response of the intestinal gut microbiota was significantly affected by the stress level to which the fish was exposed, which finally resulted in a significant discrimination of microbial composition based on phylogenetic distance matrices. As has been proposed elsewhere, these results support the hypothesis of a connection between physiological conditions (induced by stress) and nutrition. As has been shown in mammals [8] and zebrafish [30], the microbiome acts as part of the gut–brain axis, a complex network in which both parts communicate with each other via several mechanisms. Neurotransmitter released through brain-controlled glands, induced by, for example, an external stressor, can be bound by microbes and thus change, for example, nutrient absorption or pathogen defense. Conversely, members of the microbiota affect the host via afferent pathways of the vagus nerve to the central nervous system or by secreting metabolites that communicate to the immune system or passing through the intestinal barrier. Another study revealed [26] that temperature stress in combination with a soybean meal diet induces enteritis, a severe health issue in salmonid aquaculture. The question of whether stress modulates diet utilization or whether a certain diet alters coping with a stress stimulus cannot be answered up to this point. In trout line B, differences in the microbial gut composition are explained by factor stress. Our results demonstrate that the microbial gut composition of trout was not affected by the diets we provided. Further investigation needs to be conducted to elaborate on what physiological changes in the host are triggered. While the stress in trout line A interacts with diet composition, trout line B seems to be less affected by the diet, which suggests that the microbiota is able to maintain its microbial composition, not depending on which diet was fed. Similar to a study on the stress response of the brook charr microbiome [73], the genetic background of rainbow trout determines how susceptible the microbial composition is to stress, which is a trend we observed in this study. The conclusion is that the microbiome of rainbow trout fed a plant-based diet are less susceptible to an external stressor in certain breeding lines. Moreover, we already demonstrated for trout line A that the FM-based diet significantly increased the expression of particular immune markers, such as immunoglobulins D (membrane-bound and secreted) and T (membrane-bound) in contrast to the plant-based diet [35]. When exposed to stress, fish fed the FM-based diet also significantly upregulated the expression of TNFα, a proinflammatory cytokine that is upregulated in response to circulating stress hormones. With respect to the enhancing effects of plant-based diets on bacteria that have a presumably positive effect on fish health, as previously observed [10, 11, 74], it might also be possible that plant-based diets are actually supportive of rainbow trout under stress. In combination with our observations of the interaction effect of stress and diet on the intestinal microbiota in trout line A, there seems to be a strong relationship between the immune response of trout and its intestinal microbiota in response to external stimuli, which needs to be addressed in the future.

## Conclusions

Contemplating the experimental setup of this study, the most influential factor on the intestinal gut microbiota of rainbow trout is stress status. We found that the response of the microbial composition to stress is presupposed by (i) the examined trout genetic line and (ii) which diet was provided. Based on these factors, the influence of stress on the intestinal microbiota of rainbow trout changes. Although our data can demonstrate and corroborate shifts in the microbial community as a function of diet and/or stress, at this stage, we note no ability to predict how these shifts may impact the physiology of the fish or the microbiome so far. Further research related to how a stress–diet constellation affects the mechanisms at a molecular level, and especially the interaction of host and microbiome, needs to be conducted. Furthermore, the data disclosed that no effect of the aforementioned factors on alpha diversity or performance parameters was present. Additionally, the diet and stress constellation we provided had a significant impact on the abundance of two genera, namely *Cetobacterium* and *Mycoplasma*, which represent two recurrent genera within the intestinal gut microbiome of rainbow trout. The exact role of these genera in such husbandry conditions requires further investigation. Since the results of the standard performance parameters revealed no indication of an influence by stress, diet, or a diet–stress interaction, the gut microbiome composition seems likely to be a sensitive biomarker for stress exposure in aquaculture.

## Materials and methods

### Animals and experimental design

The experiment was conducted at the Fraunhofer Research Institution for Individualized and Cell-Based Medical Engineering (IMTE) (former: “Gesellschaft für Marine Aquakultur mbH” (GMA, Büsum, Germany)). All experiments involving fish handling procedures were executed with the strict permission of the animal welfare officer of the IMTE and the local authority of Schleswig-Holstein (MELUND, V 241–36,754/2018) following relevant institutional and national guidelines for the care and use of laboratory animals (German animal welfare law; TierSchG and Regulation for the Protection of Animals Used for Experimental and Other Scientific Purposes; TierSchVersV as the national implementation of Directive 2010/63/EU). The research also adhered to Aquaculture Research ethical guidelines.

The multifactorial experiment was conducted in triplicate, composed of three factors with two levels each: breeding line (A or B), diet (F or V), and stress exposure (0: no stress; 1: stress).

**Fig. 6:**
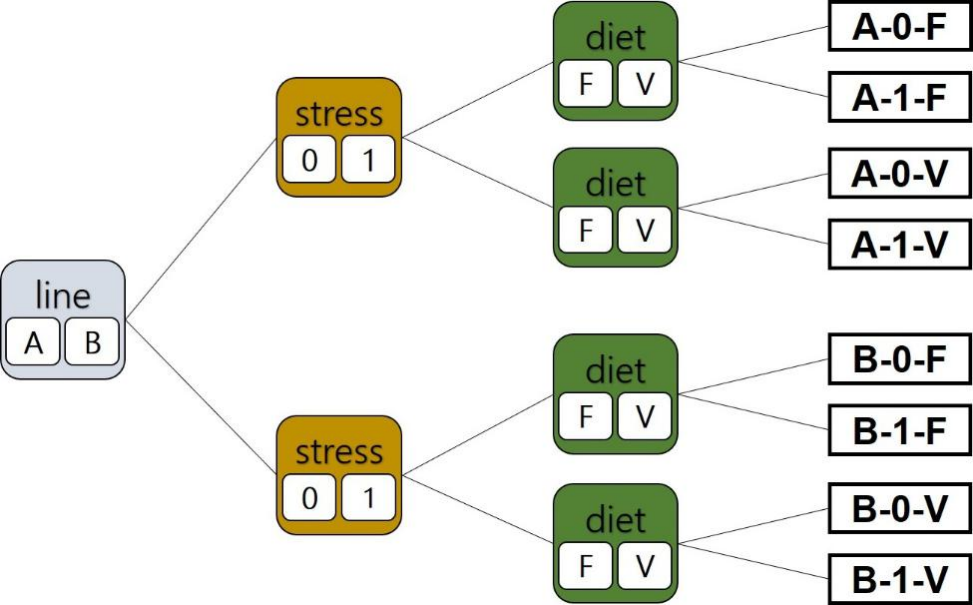
Scheme of treatments used in this study. Two different genetic trout lines were used and identified as A or B. A 60s stress impulse twice a day splits the treatments into stressed (1) and non-stressed (0) fish groups. Trout were fed either a FM-based diet (F), or a plant-based diet (V)

Fish were reared in two RASs, each consisting of a 4.0 m³ water body (20 tanks á 150 L). Breeding lines were spatially separated into two RASs to avoid epidemic risk factors. Twelve of the 20 tanks were stocked with 18 individuals from breeding line A and 12 of the 20 tanks with 16 individuals from breeding line B to assure a similar stocking density (15 kg/m³). Thus, a total of 2³ experimental groups (Fig. 6) were established in triplicates (n = 3). Tanks were stocked with female rainbow trout (*Oncorhynchus mykiss*) of either breeding line A, obtained from Forellenzucht Trostadt (Forellenzucht Trostadt GbR, Tautenhahn, Germany), which originates from Troutlodge (Bonney Lak, USA), or breeding line B, purchased from Themar Fischzuchtanlage GmbH (Themar, Germany), originating from Frédéric Cachelou (Sarrance, France). Fish were exposed to constant environmental parameters for RASs A and B, respectively, comprising of 15.17 °C ± 0.44, 15.42 °C ± 0.38 water temperature, 9.67 ± 0.69 mg l^− 1^, 9.47 ± 0.49 mg l^−1^ O_2_ concentration, pH 7.2, pH 7.16, 4.62 ppt ± 0.63, 4.56 ppt ± 0.61 salinity, 0.43 mg l^−1^ ± 0.14, 0.50 mg l^−1^ ± 0.22 Ammonium (NH_4_^+^), and 0.96 ± 0.34 mg l^−1^, 1.07 ± 0.35 mg l^− 1^nitrite (NO_2_^−^) (mean value and standard deviation) checked on a daily routine. Light was provided for 15 h per day. Prior to the trial, the fish of breeding line A were acclimatized to the rearing system for six weeks, while the fish of breeding line B were acclimatized for 12 days due to logistic issues concerning fish acquisition. The experimental trial lasted 59 days in total. During adaptation, fish feeding rates were restricted to 0.5% of total biomass per day, applying the original diets of the respective breeding facility to avoid additional feeding stress effects (commercial trout diets from BioMar for line A and Skretting for line B).

### Diets

Rainbow trout were fed two experimental, isonitrogenous, and isoenergetic diets, formulated according to commercial trout diets differing in fish protein (35% diet F, 7% diet V; Table 3) and plant-based proteins (47% diet F, 73% diet V). Fish diets were purchased from a company specialized in the production of experimental feed (Sparos Lda., Olhão, Portugal). Pellets were extruded to a size of 4 mm and fat was applied via vacuum coating. Two times per day, 1.5% of the total tank biomass was fed per hand.

### Handling

To induce handling stress and to avoid a bias in feed intake, fish were forcefully chased with aquarium nets twice a day for 60 s, two hours after each feeding event. Stressed and non-stressed fish were spatially separated within the RASs (experimental block design) to avoid random effects in the non-stressed experimental groups caused by chasing in the surrounding tanks. To prevent any skin lesions or further injuries, stress activities were conducted with high precautions to avoid direct contact with the nets. No mortalities occurred during the experiment in either treatment group. Before the start of the trial, all fish stocked into the two RASs were individually weighed (initial body weight; IBW), as well as all sampled fish at the end of the trial for the microbiome analysis and some additional samples used in another study for blood analysis (N = 240) after 59 days. Group weights of the experimental groups were determined on the starting day and on day 15, 35, and 50 to calculate the specific growth rate (SGR), feed conversion ratio (FCR), daily feed intake (DFI), protein efficiency ratio (PER), and protein retention efficiency (PRE) (Supplementary file; [35]). The last group measurements for performance parameters were acquired after 50 days to avoid additional handling of stress effects for the final sampling on day 59.

**Table 3:** Experimental diets F (FM-based) and V (plant-based). The analytical components of the diet ingredients (% of original substance) were provided by SPAROS Lda, Olhão, Portugal. Information about nutritional values and energy content was acquired at the IMTE, Büsum

Ingredient	F [%]	V [%]
Fish meal LT70^a^	35.00	7.00
Fish protein concentrate	2.50	2.50
Soy protein concentrate^b^	6.00	20.50
Wheat gluten	6.00	15.20
Corn gluten	5.00	5.00
Soybean meal 48^c^	0.00	5.00
Wheat meal	15.00	11.40
Faba beans (low tannins)	6.00	6.00
Fish oil	13.92	14.64
Rapeseed oil	9.28	9.76
Vitamin & Mineral Premixtures INVIVO 1%	1.00	1.00
Vitamin C^d^	0.05	0.05
Vitamin E^e^	0.05	0.05
Antioxidant	0.20	0.20
Monocalcium phosphate	0.00	1.00
L-lysine	0.00	0.10
L-tryptophan	0.00	0.10
DL-methionine	0.00	0.50
**Total**	100.00	100.00
**Nutritional composition (as fed basis)**		
Water	5.58	5.22
Crude ash	7.74	4.85
Crude protein	41.75	42.66
Crude fat	26.50	26.48
Carbohydrate^f^	18.43	20.79
Gross Energy [MJ kg^− 1^]	23.54	23.92

^a^Peruvian fishmeal LT: 670 g kg^− 1^ crude protein (CP), 90 g kg^− 1^ crude fat (CF), EXALMAR, Peru.

^b^Soycomil PC: 630 g kg − 1 CP, < 10 g kg − 1 CF, ADM, The Netherlands.

^c^Solvent extracted dehulled soybean meal: 480 g kg − 1 CP, 26 g kg − 1 CF, SORGAL SA, Portugal.

^d^Vitamin C: >35% sodium and calcium salts of ascorbyl-2-phosphate, Lutavit C35, BASF, Germany.

^e^Vitamin E: >50% DL-alpha-tocopheryl acetate, Lutavit E50, BASF, Germany.

^f^Carbohydrate = 100 – (water – crude ash – crude protein – crude fat).

### Sample collection

Before the start of the trial, 21 fish from each breeding line and at the end of the trial (day 59), in total 169 fish (21 fish/treatment, 7 fish/tank), were randomly sampled for microbiome analysis as follows: animals were anesthetized with a blow on the head and killed afterwards by cutting the gill vein. By placing fish on clean surfaces, the gastrointestinal tract was dissected using sterile scissors and scalpels. Feces were collected by squeezing the entire intestinal tissue posterior to the stomach toward the anus. Fecal samples were stored immediately on dry ice in 5 ml sterile tubes.

### DNA isolation, library preparation, and sequencing

A total of 250 mg of pre-homogenized gut content was mixed with 1 ml InhibitEx buffer in 0.70 mm Garnet Bead tubes. Lysis of bacterial cells was achieved in a SpeedMill PLUS (Analytik Jena GmbH, Jena, DE) at a high speed for 45 s. After an incubation time of 5 min at 550 rpm by 95 °C, lysed cells were pelletized for 1 min at 20.000 rcf. Total bacterial DNA culled from fish gut contents was subsequently extracted using 200 µl supernatant as input for QIAmp® DNA fast stool mini kit (Qiagen, USA, Cat. no. 51,604), which was conducted according to the manufacturer’s instructions on a QIAcube® automation machine (Qiagen, USA). Additionally, 12 extraction blanks were included to obtain possible contamination during DNA preparation.

For sequencing, a one-step polymerase chain reaction (PCR) amplification of the V3–V4 region of the 16S rRNA gene was performed using forward and reverse primer 341 F ‘CCTACGGGAGGCAGCAG’ and 805R ‘GGACTACHVGGGTWTCTAAT’ [75], respectively, in a dual-barcoding approach. A final volume of 26 µl comprised of 5.0 µl 5X Phusion HF buffer, 0.5 µl dNTP (10 mM), 0.3 µl Phusion Hot Start II Polymerase (2 U/µl; Thermo Fisher Scientific), 9.2 H_2_O, 4 µl of each primer (100 µM), and 3 µl microbial DNA template were prepared. Each PCR reaction plate included a negative control with nuclease-free water to account for contamination and a reaction employed with a mock community (ZymoBIOMICS Microbial Community Standard, Cat. no. D6305) as a positive control composed of eight bacterial isolates with defined abundances to verify adequate performance. Following an initial denaturation temperature of 98 °C for 30 s, 30 cycles were carried out as follows: 98 °C (0:09 min), 55 °C (1:00 min) annealing temperature, and 72 °C (1:30 min) extension temperature. After cycling, the amplificants were extended with a final round at 72 °C for 10:00 min. The expected fragment size of 550 bp was verified via gel electrophoresis on a 2.0% agarose gel.

Sequencing of the V3–V4 region from the 16S rRNA gene amplicons of fish gut contents, positive and negative controls (in total 294 sequencing samples) was executed at the Institute of Clinical Molecular Biology (IKMB, Kiel University) on an Illumina MiSeq platform (Illumina Inc., San Diego, CA, USA) using MiSeq Reagent kit v3 according to the manufacturer’s specification and Trautmann et al. [76], ending up with 2 × 300 bp paired-end reads. Raw sequences obtained during the study are stored at the NCBI Sequence Read Archive (SRA) and can be accessed via the SRA accession number SRP355371 or the BioProject ID PRJNA797926.

### Bioinformatics

Demultiplexed reads exempted from sequencing adapters were provided by the sequencing facility as FASTQ files, of which read quality was examined using FastQC [77] and MultiQC [78]. FASTQ files were imported into Quantitative Insights Into Microbial Ecology 2 (Qiime2 2021.2.0 [79]) where the following processing was performed. The Cutadapt plugin [80] was used to trim any leftover primers and/or spacers. To denoise, filter for low-quality reads and chimeras, and merge paired-end reads, the integrated DADA2 plugin [81] was run with truncation parameters of 270 for forward reads, 247 for reverse reads, and a truncation quality cutoff of 5. The outcome resulted in a feature table based on the ASVs and their corresponding representative sequences. Features with an occurrence in less than two samples or an overall frequency of less than 16 were discarded using the filter-features command of the feature-table plugin. To assign taxonomy to ASVs, a naïve Bayes classifier was trained against a SILVA 99% database (release 138), with regions of interest extracted from full-length sequences using the corresponding primer V3–V4 and the feature-classifier plug-in. Reads from the SILVA database were extracted to generate custom reference sequences. Features classified as *Cyanobacteria*, assumed to derive from plant chloroplasts, and mitochondrial origins were excluded from further analysis. Only ASVs classified as bacteria and identified at least to the phylum level were kept. Based on the extraction controls, five extraction batches were excluded from further analysis due to contamination effects. A phylogenetic tree was generated using a *de novo* approach by conducting a multiple alignment using a fast Fourier transform via the *qiime phylogeny* plugin. Mock community samples were separated from the dataset and analyzed for accurate expected abundance according to the manufacturer.

The following analysis was performed using RStudio and the R package *phyloseq* [82]. For alpha diversity analysis, richness was estimated using observed features, Shannon diversity, and Simpson index, which additionally included evenness. All parameters were estimated based on the genus level. To compare how many taxa are shared among samples (beta-diversity), unweighted and weighted UniFrac distances were assessed using the R package *vegan* [83] and visualized using NMDS.

### Statistics

Statistical evaluation of the data was executed using the computation software R (2021). Growth performance parameters, alpha diversity, and relative abundances of taxa evaluation began with the definition of an appropriate statistical mixed model [84, 85]. Both breeding lines were tested in separate models. The particular model included diet and stress, as well as all their interaction terms (two-fold), as fixed factors. The tanks were regarded as a random factor. The residuals were assumed to be approximately normally distributed and heteroscedastic. These assumptions are based on graphical residual analysis. Based on this model, a pseudo R² was calculated [86] and a two-way ANOVA was conducted, followed by multiple contrast tests [87] in order to compare the several levels of the influence factors. The initial samples were tested against the treatments (sampling time) for alpha diversity and abundance data in a separate model while using the same parameters.

Multivariate analysis of beta diversity in rainbow trout gut microbiome communities was applied to weighted and unweighted UniFrac distances using the adonis function from the *vegan* package with 999 permutations, with diet and stress as interaction factors. Pairwise PERMANOVA was executed with pseudofactor treatment as a factor and no p-value adjustment using the R package *RVAideMemoire*. Instead, the required comparisons were chosen, and p-values were adjusted manually using the method ‘holm.’ Multivariate homogeneity of group dispersions (betadisper, *vegan*) using treatments as pseudofactors was conducted.

LEfSe [88] was applied using the R package *microbiomeMarker* to identify bacteria that contribute the most to the differences in community by performing LDA scoring to estimate the effect size (threshold ≥ 3.5) with treatment as a pseudofactor and non-parametric Kruskal–Wallis test using the mean values. All p-values obtained by LEfSe were corrected for multiple comparisons using the Benjamani–Hochberg false discovery rate method.

## Electronic supplementary material

Below is the link to the electronic supplementary material.

Supplementary Material 1. S1 to S5. Performance parameters and additional figures (S1 to S5)

Supplementary Material 2. T1 to T8. Relative abundance data and statistical results from alpha and beta diversity (T1–T8)

## Data Availability

Raw sequencing files are available from the NCBI Sequence Read Archive (SRA) via Study ID SRP355371 within BioProject PRJNA797926.

## References

[1] FAO. The state of world fisheries and aquaculture 2020: stustainability in action. FAO; 2020.

[2] Gatlin DM, Barrows FT, Brown P, Dabrowski K, Gaylord TG, Hardy RW (2007). Expanding the utilization of sustainable plant products in aquafeeds: a review. Aquaculture Res.

[3] Wong S, Waldrop T, Summerfelt S, Davidson J, Barrows F, Kenney PB (2013). Aquacultured rainbow trout (Oncorhynchus mykiss) possess a large core intestinal microbiota that is resistant to variation in diet and rearing density. Appl Environ Microbiol.

[4] Makkar HP, Tran G, Heuzé V, Ankers P (2014). State-of-the-art on use of insects as animal feed. Anim Feed Sci Technol.

[5] Naylor RL, Hardyb RW, Bureauc DP, Chiua A, Elliottd M, Farrelle AP et al. Correction for Ma, Selective activation of the M1 muscarinic acetylcholine receptor achieved by allosteric potentiation. *Proceedings of the National Academy of Sciences*. 2009;42:18040. 10.1073/pnas.0900903106 PMC2732705 19717450

[6] David LA, Maurice CF, Carmody RN, Gootenberg DB, Button JE, Wolfe BE (2014). Diet rapidly and reproducibly alters the human gut microbiome. Nature.

[7] Michl SC, Ratten J-M, Beyer M, Hasler M, LaRoche J, Schulz C (2017). The malleable gut microbiome of juvenile rainbow trout (Oncorhynchus mykiss): Diet-dependent shifts of bacterial community structures. PLoS ONE.

[8] Foster JA, Rinaman L, Cryan JF. Stress & the gut-brain axis: regulation by the microbiome. Neurobiol Stress. 2017:124–36. 10.1016/j.ynstr.2017.03.001 PMC5736941 29276734

[9] Michl SC, Beyer M, Ratten J-M, Hasler M, LaRoche J, Schulz C (2019). A diet-change modulates the previously established bacterial gut community in juvenile brown trout (Salmo trutta). Sci Rep.

[10] Desai AR, Links MG, Collins SA, Mansfield GS, Drew MD, van Kessel AG et al. Effects of plant-based diets on the distal gut microbiome of rainbow trout (Oncorhynchus mykiss). Aquaculture. 2012:134–42.

[11] Araújo C, Muñoz-Atienza E, Nahuelquín Y, Poeta P, Igrejas G, Hernández PE et al. Inhibition of fish pathogens by the microbiota from rainbow trout (Oncorhynchus mykiss, Walbaum) and rearing environment. Anaerobe. 2015:7–14. 10.1016/j.anaerobe.2014.11.001 25464142

[12] Askarian F, Zhou Z, Olsen RE, Sperstad S, Ringø E. Culturable autochthonous gut bacteria in Atlantic salmon (*Salmo salar* L.) fed diets with or without chitin. Characterization by 16S rRNA gene sequencing, ability to produce enzymes and in vitro growth inhibition of four fish pathogens. Aquaculture. 2012:1–8.

[13] Ringø E, Løvmo L, Kristiansen M, Bakken Y, Salinas I, Myklebust R (2010). Lactic acid bacteria vs. pathogens in the gastrointestinal tract of fish: a review. Aquac Res.

[14] Ingerslev H-C, von Gersdorff Jørgensen L, Lenz Strube M, Larsen N, Dalsgaard I, Boye M et al. The development of the gut microbiota in rainbow trout (Oncorhynchus mykiss) is affected by first feeding and diet type. Aquaculture. 2014:24–34.

[15] Villasante A, Ramírez C, Rodríguez H, Dantagnan P, Hernández A, Figueroa E et al. Dietary carbohydrate-to-protein ratio influences growth performance, hepatic health and dynamic of gut microbiota in atlantic salmon (Salmo salar). Anim Nutr. 2022:261–79. 10.1016/j.aninu.2022.04.003 PMC9234083 35785253

[16] Liu H, Guo X, Gooneratne R, Lai R, Zeng C, Zhan F et al. The gut microbiome and degradation enzyme activity of wild freshwater fishes influenced by their trophic levels. Sci Rep. 2016:24340. 10.1038/srep24340 PMC4829839 27072196

[17] Roeselers G, Mittge EK, Stephens WZ, Parichy DM, Cavanaugh CM, Guillemin K (2011). Evidence for a core gut microbiota in the zebrafish. ISME J.

[18] Gajardo K, Rodiles A, Kortner TM, Krogdahl Ã, Bakke AM, Merrifield DL et al. A high-resolution map of the gut microbiota in Atlantic salmon (Salmo salar): a basis for comparative gut microbial research. Sci Rep. 2016:30893. 10.1038/srep30893 PMC4971465 27485205

[19] Michl SC, Weis B, Hutchings JA, Schulz C. Plastic responses by wild brown trout (*Salmo trutta*) to plant-based diets. Aquaculture. 2017:19–28.

[20] Mansfield GS, Desai AR, Nilson SA, van Kessel AG, Drew MD, Hill JE (2010). Characterization of rainbow trout (Oncorhynchus mykiss) intestinal microbiota and inflammatory marker gene expression in a recirculating aquaculture system. Aquaculture.

[21] Navarrete P, Magne F, Araneda C, Fuentes P, Barros L, Opazo R (2012). PCR-TTGE analysis of 16S rRNA from rainbow trout (Oncorhynchus mykiss) gut microbiota reveals host-specific communities of active bacteria. PLoS ONE.

[22] Huyben D, Vidaković A, Werner Hallgren S, Langeland M. High-throughput sequencing of gut microbiota in rainbow trout (Oncorhynchus mykiss) fed larval and pre-pupae stages of black soldier fly (Hermetia illucens). Aquaculture. 2019:485–91.

[23] Huyben D, Chiasson M, Lumsden JS, Pham PH, Chowdhury MAK. Dietary microencapsulated blend of organic acids and plant essential oils affects intestinal morphology and microbiome of rainbow trout (Oncorhynchus mykiss). Microorganisms. 2021;10. 10.3390/microorganisms9102063 PMC8537560 34683384

[24] Huyben D, Nyman A, Vidaković A, Passoth V, Moccia R, Kiessling A et al. Effects of dietary inclusion of the yeasts Saccharomyces cerevisiae and Wickerhamomyces anomalus on gut microbiota of rainbow trout. Aquaculture. 2017:528–37.

[25] Perry WB, Lindsay E, Payne CJ, Brodie C, Kazlauskaite R (2020). The role of the gut microbiome in sustainable teleost aquaculture. Proc Biol Sci.

[26] Urn PA, Schrama JW, Rombout J, Obach A, Jensen L, Koppe W (2008). Soybean meal-induced enteritis in Atlantic salmon (Salmo salar L.) at different temperatures. Aquacult Nutr.

[27] Huyben D, Sun L, Moccia R, Kiessling A, Dicksved J, Lundh T (2018). Dietary live yeast and increased water temperature influence the gut microbiota of rainbow trout. J Appl Microbiol.

[28] Brown RM, Wiens GD, Salinas I. Analysis of the gut and gill microbiome of resistant and susceptible lines of rainbow trout (Oncorhynchus mykiss). Fish Shellfish Immunol. 2019:497–506. 10.1016/j.fsi.2018.11.079 PMC8040288 30513381

[29] Kelly C, Salinas I. Under pressure: interactions between commensal microbiota and the teleost immune system. Front Immunol. 2017:559. 10.3389/fimmu.2017.00559 PMC5430139 28555138

[30] Davis DJ, Bryda EC, Gillespie CH, Ericsson AC. Microbial modulation of behavior and stress responses in zebrafish larvae. Behav Brain Res. 2016:219–27. 10.1016/j.bbr.2016.05.040 PMC6423445 27217102

[31] Galley JD, Nelson MC, Yu Z, Dowd SE, Walter J, Kumar PS et al. Exposure to a social stressor disrupts the community structure of the colonic mucosa-associated microbiota. BMC Microbiol. 2014:189. 10.1186/1471-2180-14-189 PMC4105248 25028050

[32] Partrick KA, Chassaing B, Beach LQ, McCann KE, Gewirtz AT, Huhman KL. Acute and repeated exposure to social stress reduces gut microbiota diversity in syrian hamsters. Behav Brain Res. 2018:39–48. 10.1016/j.bbr.2018.02.005 PMC6246037 29474810

[33] Uren Webster TM, Rodriguez-Barreto D, Consuegra S, Garcia de Leaniz C. Cortisol-related signatures of stress in the fish microbiome. Front Microbiol. 2020:1621. 10.3389/fmicb.2020.01621 PMC7381252 32765459

[34] Callet T, Dupont-Nivet M, Danion M, Burel C, Cluzeaud M, Surget A et al. Why do some rainbow trout genotypes grow better with a complete plant-based diet? Transcriptomic and physiological analyses on three isogenic lines. Front Physiol. 2021:732321. 10.3389/fphys.2021.732321 PMC8440921 34539452

[35] Seibel H, Krassilnikova K, Fichtner-Grabowski F-T, Rebl A, Schulz C, Hornburg SC (2022). Interactions of plant‐based feeding and handling stress on the expression of selected immune markers in rainbow trout (Oncorhynchus mykiss). Aquac Res.

[36] Ingerslev H-C, Strube ML, Jørgensen LvG, Dalsgaard I, Boye M, Madsen L (2014). Diet type dictates the gut microbiota and the immune response against Yersinia ruckeri in rainbow trout (Oncorhynchus mykiss). Fish Shellfish Immunol.

[37] Llewellyn MS, Boutin S, Hoseinifar SH, Derome N (2014). Teleost microbiomes: the state of the art in their characterization, manipulation and importance in aquaculture and fisheries. Front Microbiol.

[38] Bruce TJ, Neiger RD, Brown ML (2018). Gut histology, immunology and the intestinal microbiota of rainbow trout, Oncorhynchus mykiss (Walbaum), fed process variants of soybean meal. Aquac Res.

[39] Terova G, Rimoldi S, Ascione C, Gini E, Ceccotti C, Gasco L (2019). Rainbow trout (Oncorhynchus mykiss) gut microbiota is modulated by insect meal from Hermetia illucens prepupae in the diet. Rev Fish Biol Fisheries.

[40] Colston TJ, Jackson CR (2016). Microbiome evolution along divergent branches of the vertebrate tree of life: what is known and unknown. Mol Ecol.

[41] Lyons PP, Turnbull JF, Dawson KA, Crumlish M (2017). Phylogenetic and functional characterization of the distal intestinal microbiome of rainbow trout Oncorhynchus mykiss from both farm and aquarium settings. J Appl Microbiol.

[42] Ghanbari M, Kneifel W, Domig KJ. A new view of the fish gut microbiome: advances from next-generation sequencing. Aquaculture 2015;Suppl 5:464–75.

[43] Murphy CL, Biggerstaff J, Eichhorn A, Ewing E, Shahan R, Soriano D (2021). Genomic characterization of three novel Desulfobacterota classes expand the metabolic and phylogenetic diversity of the phylum. Environ Microbiol.

[44] Hines IS, Ferguson CS, Bushman TJ, Gatlin DM, Jensen RV, Smith SA (2021). Impact of a yeast-based dietary supplement on the intestinal microbiome of rainbow trout, Oncorhynchus mykiss. Aquac Res.

[45] Rimoldi S, Terova G, Ascione C, Giannico R, Brambilla F (2018). Next generation sequencing for gut microbiome characterization in rainbow trout (Oncorhynchus mykiss) fed animal by-product meals as an alternative to fishmeal protein sources. PLoS ONE.

[46] Boutin S, Bernatchez L, Audet C, Derôme N (2013). Network analysis highlights complex interactions between pathogen, host and commensal microbiota. PLoS ONE.

[47] Li T, Long M, Gatesoupe F-J, Zhang Q, Li A, Gong X (2015). Comparative analysis of the intestinal bacterial communities in different species of carp by pyrosequencing. Microb Ecol.

[48] van Kessel MA, Dutilh BE, Neveling K, Kwint MP, Veltman JA, Flik G et al. Pyrosequencing of 16S rRNA gene amplicons to study the microbiota in the gastrointestinal tract of carp (Cyprinus carpio L.). AMB Expr. 2011;1. 10.1186/2191-0855-1-41 PMC3226434 22093413

[49] He M, Li X, Poolsawat L, Guo Z, Yao W, Zhang C (2020). Effects of fish meal replaced by fermented soybean meal on growth performance, intestinal histology and microbiota of largemouth bass (Micropterus salmoides). Aquacult Nutr.

[50] Larsen AM, Mohammed HH, Arias CR (2014). Characterization of the gut microbiota of three commercially valuable warmwater fish species. J Appl Microbiol.

[51] Finegold SM, Vaisanen M-L, Molitoris DR, Tomzynski TJ, Song Y, Liu C (2003). Cetobacterium somerae sp. nov. from human feces and emended description of the genus Cetobacterium. Syst Appl Microbiol.

[52] Tsuchiya C, Sakata T, Sugita H (2007). Novel ecological niche of Cetobacterium somerae, an anaerobic bacterium in the intestinal tracts of freshwater fish. Lett Appl Microbiol.

[53] Silva FCdP, Nicoli JR, Zambonino-Infante JL, Kaushik S, Gatesoupe F-J (2011). Influence of the diet on the microbial diversity of faecal and gastrointestinal contents in gilthead sea bream (Sparus aurata) and intestinal contents in goldfish (Carassius auratus). FEMS Microbiol Ecol.

[54] Lyons PP, Turnbull JF, Dawson KA, Crumlish M (2017). Exploring the microbial diversity of the distal intestinal lumen and mucosa of farmed rainbow trout Oncorhynchus mykiss (Walbaum) using next generation sequencing (NGS). Aquac Res.

[55] Gatesoupe FJ, Fauconneau B, Deborde C, Madji Hounoum B, Jacob D, Moing A (2018). Intestinal microbiota in rainbow trout, Oncorhynchus mykiss, fed diets with different levels of fish-based and plant ingredients: a correlative approach with some plasma metabolites. Aquacult Nutr.

[56] Parks DH, Chuvochina M, Waite DW, Rinke C, Skarshewski A, Chaumeil P-A (2018). A standardized bacterial taxonomy based on genome phylogeny substantially revises the tree of life. Nat Biotechnol.

[57] Lyons PP, Turnbull JF, Dawson KA, Crumlish M (2017). Effects of low-level dietary microalgae supplementation on the distal intestinal microbiome of farmed rainbow trout Oncorhynchus mykiss (Walbaum). Aquac Res.

[58] Lowrey L, Woodhams DC, Tacchi L, Salinas I (2015). Topographical mapping of the rainbow trout (Oncorhynchus mykiss) microbiome reveals a diverse bacterial community with antifungal properties in the skin. Appl Environ Microbiol.

[59] Villasante A, Ramirez C, Catalán N, Romero J (2017). First report of swim bladder-associated microbiota in rainbow trout (Oncorhynchus mykiss). Microbes Environ.

[60] Rasmussen JA, Villumsen KR, Duchêne DA, Puetz LC, Delmont TO, Sveier H (2021). Genome-resolved metagenomics suggests a mutualistic relationship between Mycoplasma and salmonid hosts. Commun Biol.

[61] Cheaib B, Yang P, Kazlauskaite R, Lindsay E, Heys C, Dwyer T et al. Genome erosion and evidence for an intracellular niche - exploring the biology of mycoplasmas in Atlantic salmon. Aquaculture. 2021:736772. 10.1016/j.aquaculture.2021.736772 PMC8192413 34471330

[62] Huyben D, Roehe BK, Bekaert M, Ruyter B, Glencross B. Dietary lipid: protein ratio and n-3 long-chain polyunsaturated fatty acids alters the gut microbiome of Atlantic salmon under hypoxic and normoxic conditions. Front Microbiol. 2020:589898. 10.3389/fmicb.2020.589898 PMC7785582 33424792

[63] Llewellyn MS, McGinnity P, Dionne M, Letourneau J, Thonier F, Carvalho GR (2016). The biogeography of the atlantic salmon (Salmo salar) gut microbiome. ISME J.

[64] Holben WE, Williams P, Gilbert MA, Saarinen M, Särkilahti LK, Apajalahti JHA (2002). Phylogenetic analysis of intestinal microflora indicates a novel Mycoplasma phylotype in farmed and wild salmon. Microb Ecol.

[65] Meseguer MA, Alvarez A, Rejas MT, Sánchez C, Pérez-Díaz JC, Baquero F (2003). Mycoplasma pneumoniae: a reduced-genome intracellular bacterial pathogen. Infect Genet Evol.

[66] Rimoldi S, Gini E, Iannini F, Gasco L, Terova G. The effects of dietary insect meal from Hermetia illucens Prepupae on Autochthonous gut microbiota of rainbow trout (Oncorhynchus mykiss). *Animals (Basel)*. 2019;4. 10.3390/ani9040143 PMC6523354 30987067

[67] Terova G, Gini E, Gasco L, Moroni F, Antonini M, Rimoldi S (2021). Effects of full replacement of dietary fishmeal with insect meal from Tenebrio molitor on rainbow trout gut and skin microbiota. J Anim Sci Biotechnol.

[68] Al-Hisnawi A, Rodiles A, Rawling MD, Castex M, Waines P, Gioacchini G (2019). Dietary probiotic Pediococcus acidilactici MA18/5 M modulates the intestinal microbiota and stimulates intestinal immunity in rainbow trout (Oncorhynchus mykiss). J World Aquacult Soc.

[69] Bozzi D, Rasmussen JA, Carøe C, Sveier H, Nordøy K, Gilbert MTP (2021). Salmon gut microbiota correlates with disease infection status: potential for monitoring health in farmed animals. Anim Microbiome.

[70] Sahandi J, Jafaryan H, Soltani M, Ebrahimi P (2019). The use of two Bifidobacterium strains enhanced growth performance and nutrient utilization of rainbow trout (Oncorhynchus mykiss) fry. Probiotics Antimicrob Proteins.

[71] Merrifield DL, Balcázar JL, Daniels C (2014). Indigenous lactic acid bacteria in fish and crustaceans. Aquaculture Nutrition: Gut Health, Probiotics and Prebiotics.

[72] Kokou F, Sasson G, Nitzan T, Doron-Faigenboim A, Harpaz S, Cnaani A et al. Host genetic selection for cold tolerance shapes microbiome composition and modulates its response to temperature. Elife. 2018. 10.7554/eLife.36398 PMC6277203 30454554

[73] Crespel A, Bernatchez L, Garant D, Audet C (2011). Quantitative genetic analysis of the physiological stress response in three strains of brook charr Salvelinus fontinalis and their hybrids. J Fish Biol.

[74] Merrifield DL, Erik R, Myklebust R, Ring E. Dietary effect of soybean (glycine max) products on gut histology and microbiota of fish. in *Soybean and Nutrition*, H. El-Shemy, Ed., InTech. 2011.

[75] Klindworth A, Pruesse E, Schweer T, Peplies J, Quast C, Horn M (2013). Evaluation of general 16S ribosomal RNA gene PCR primers for classical and next-generation sequencing-based diversity studies. Nucleic Acids Res.

[76] Trautmann T, Bang C, Franke A, Vincent D, Reinshagen K, Boettcher M. The impact of oral sodium chloride supplementation on thrive and the intestinal microbiome in neonates with small bowel ostomies: a prospective cohort study. Front Immunol. 2020:1421. 10.3389/fimmu.2020.01421 PMC7365880 32754153

[77] Andrews S. FastQC: A quality control tool for high throughput sequence data [Online].2010, http://www.bioinformatics.babraham.ac.uk/projects/fastqc/.

[78] Ewels P, Magnusson M, Lundin S (2016). MultiQC: summarize analysis results for multiple tools and samples in a single report. Bioinformatics.

[79] Bolyen E, Rideout JR, Dillon MR, Bokulich NA, Abnet CC, Al-Ghalith GA (2019). Reproducible, interactive, scalable and extensible microbiome data science using QIIME 2. Nat Biotechnol.

[80] Martin M (2011). Cutadapt removes adapter sequences from high-throughput sequencing reads. EMBnet j.

[81] Callahan BJ, McMurdie PJ, Rosen MJ, Han AW, Johnson AJA, Holmes SP (2016). DADA2: high-resolution sample inference from Illumina amplicon data. Nat Methods.

[82] McMurdie PJ, Holmes S (2013). Phyloseq: an R package for reproducible interactive analysis and graphics of microbiome census data. PLoS ONE.

[83] Oksanen J, Blanchet FG, Friendly M, Kindt R, Legendre P, McGlinn D et al. Vegan: Community Ecology Package. 2020, https://CRAN.R-project.org/package=vegan.

[84] Pinheiro J, Bates D. Mixed-effects models in S and S-PLUS. Springer science & business media. 2006.

[85] Carroll RJ, Ruppert D. Transformation and weighting in Regression. 1988.

[86] Nakagawa S, Schielzeth H, O’Hara RB (2013). A general and simple method for obtaining R2 from generalized linear mixed-effects models. Methods Ecol Evol.

[87] Hothorn T, Bretz F, Westfall P (2008). Simultaneous inference in general parametric models. Biom J.

[88] Segata N, Izard J, Waldron L, Gevers D, Miropolsky L, Garrett WS (2011). Metagenomic biomarker discovery and explanation. Genome Biol.

