# Supplementary material for: The microbiota knows: handling-stress and diet transform the microbial landscape in the gut content of rainbow trout in RAS
Source: Anim Microbiome. 2023 Jun 29;5:33. doi: 10.1186/s42523-023-00253-9 (PMC10311755; doi:10.1186/s42523-023-00253-9)
Supplement: Supplementary file 1 — Supplementary Material 1. S1 to S5. Performance parameters and additional figures (S1 to S5) [file 42523_2023_253_MOESM1_ESM.pdf]

The microbiota knows: Handling-stress and diet transform the microbial landscape in the gut content of rainbow trout in RAS

Marvin Suhr[1], Finn-Thorbjörn Fichtner-Grabowski[1], Henrike Seibel[2], Corinna Bang[3], Andre Franke[3], Carsten Schulz[2,4], Stéphanie C Hornburg[1]

[1]Institute of Animal Nutrition and Physiology, Christian-Albrechts-University Kiel, Hermann-Rodewald-Straße 9, 24118 Kiel, Germany

[2]Fraunhofer Research Institution for Individualized and Cell-Based Medical Engineering (IMTE), Hafentörn 3, 25761 Büsum, Germany

[3]Institute of Clinical Molecular Biology, Christian-Albrechts-University Kiel, Rosalind-Franklin-Str. 12, 24105 Kiel, Germany

[4]Institute of Animal Breeding and Husbandry, Team Marine Aquaculture, Christian-Albrechts-University Kiel, Hermann-Rodewald-Straße 6, 24118 Kiel, Germany

* Corresponding authors: Marvin Suhr (suhr@aninut.uni-kiel.de), Stéphanie Céline Hornburg (hornburg@aninut.uni-kiel.de)

Performance parameters

Specific growth rate (SGR), feed conversion ratio (FCR), daily feed intake (DFI), protein efficiency ratio (PER) and protein retention efficiency (PRE) (performance parameters) where calculated based on group measures by the following equations:

$$SGR = [\ln(FGW) - \ln(IGW)] / t * 100$$

$$FCR = (F_t / 1000) / (FGW - IGW)$$

$$DFI = SGR * FCR$$

$$PER = (FGW - IGW) * 1000 / CP_t$$

$$PRE = CP_g / CP_t * 100$$

FWG: final group weight (kg), IGW: initial group weight (kg), t: 59 days, F_t: total feed intake (g), CP_t: total crude protein intake (g), CP_g: total gained body protein (g)

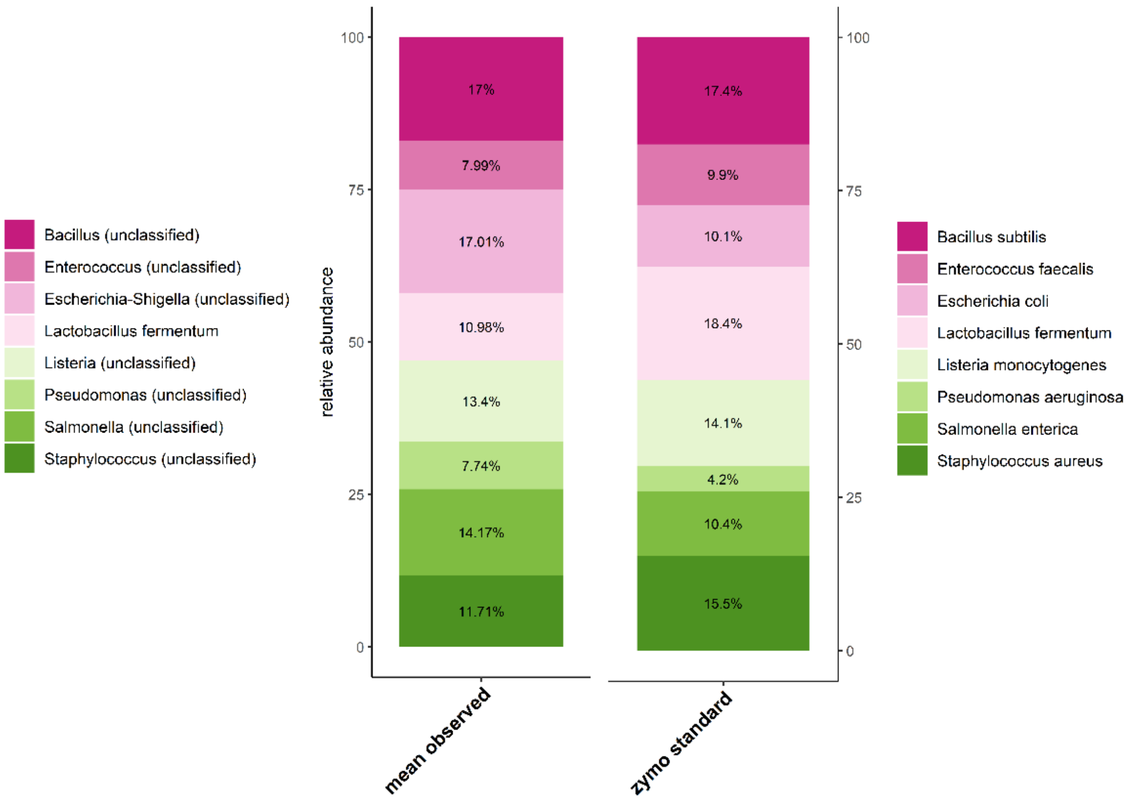

Figure S1 *Left bar plot (mean observed) shows mean relative abundance of three mock community probes sequenced parallel to the trial samples. Right bar plot (zymo standard) shows relative abundance given by the manufacturer (Zymo).*

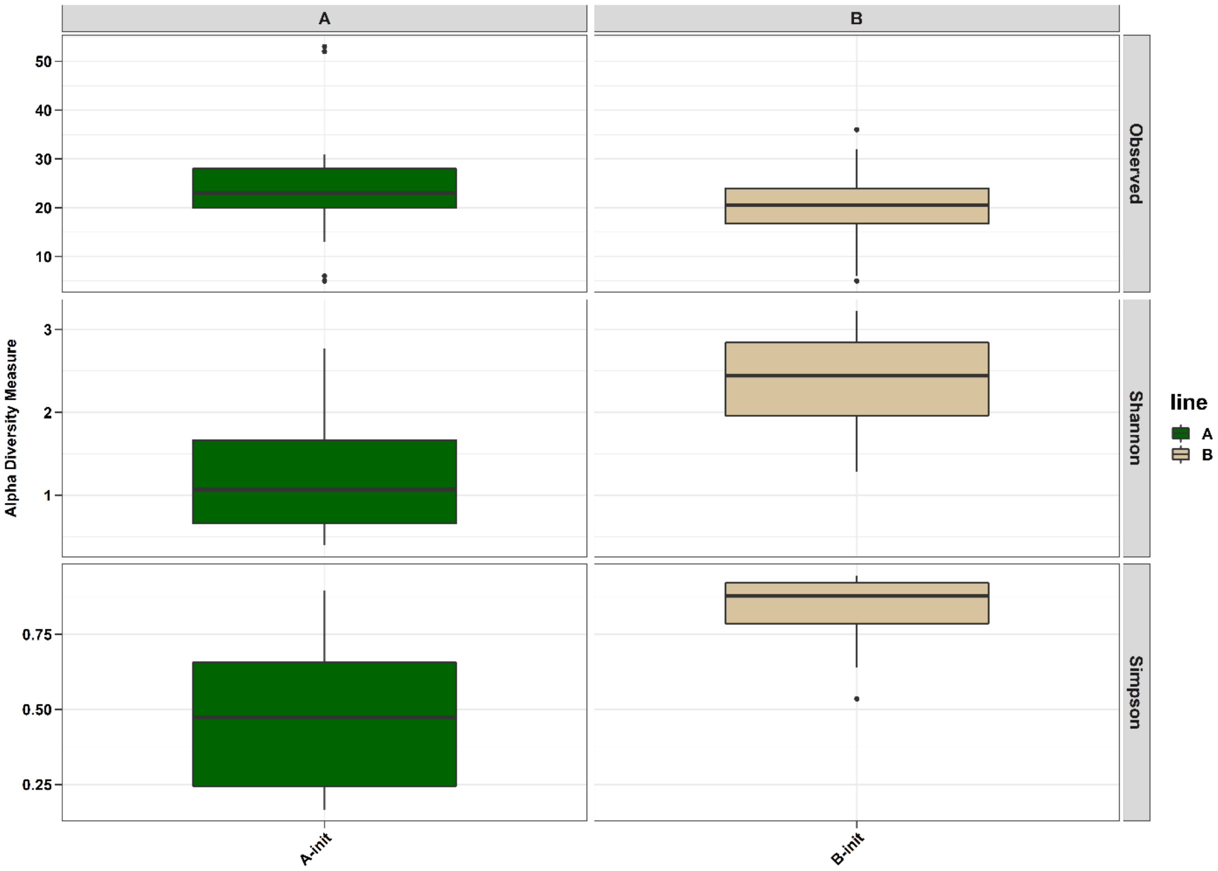

Figure S2 *Alpha diversity indices Observed ASVs, Shannon diversity and Simpson of initial sampling from trout line A and B.*

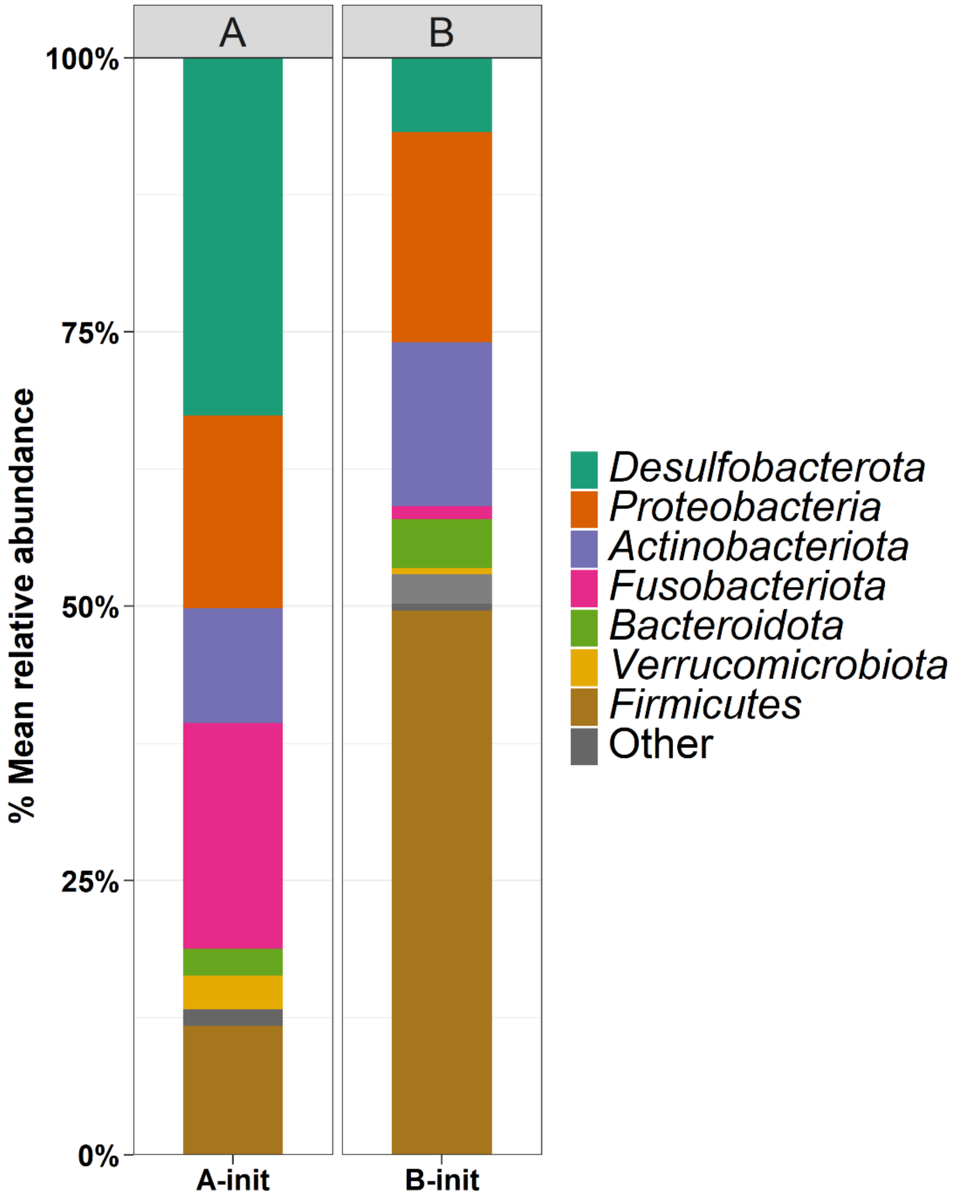

Figure S3 *Mean relative abundance (%) at the phylum level of the intestinal microbiota from both trout lines past adaptation time and prior to the experiment. Genera with a representation of 1.5 % were pooled in category 'Other'. The arrangement of the bars is based on abundance, except for the most abundant phyla, which is placed at the bottom for legibility. The mean value joins data on each individual fish for the corresponding trout line.*

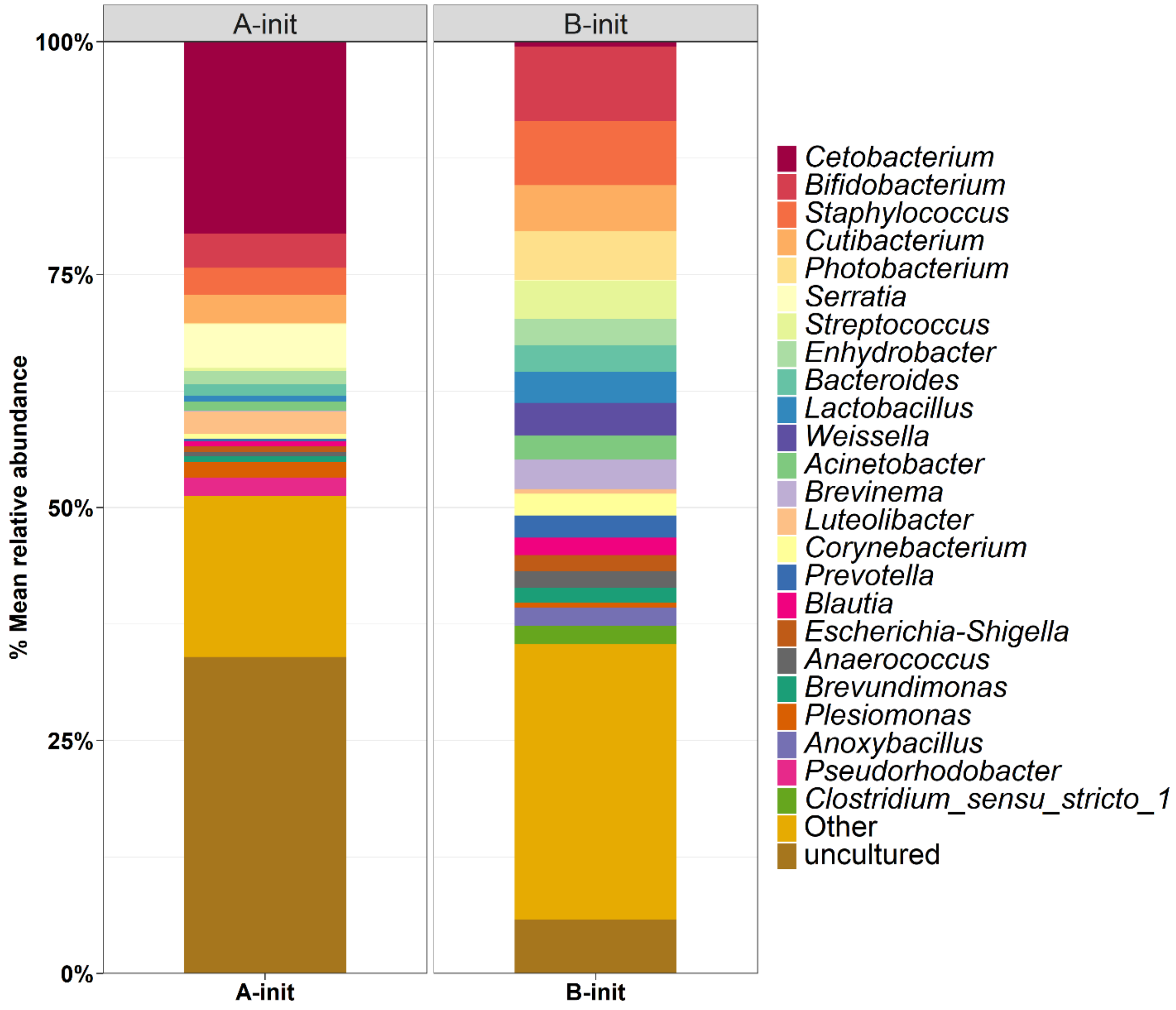

***Figure S4** Mean relative abundance (%) at the genus level of the intestinal microbiota from both trout lines past adaptation time and prior to the experiment. Genera with a representation of 1.5 % were pooled in category 'Other'. The arrangement of the bars is based on abundance, except for the most abundant phyla, which is placed at the bottom for legibility. The mean value joins data on each individual fish for the corresponding trout line.*

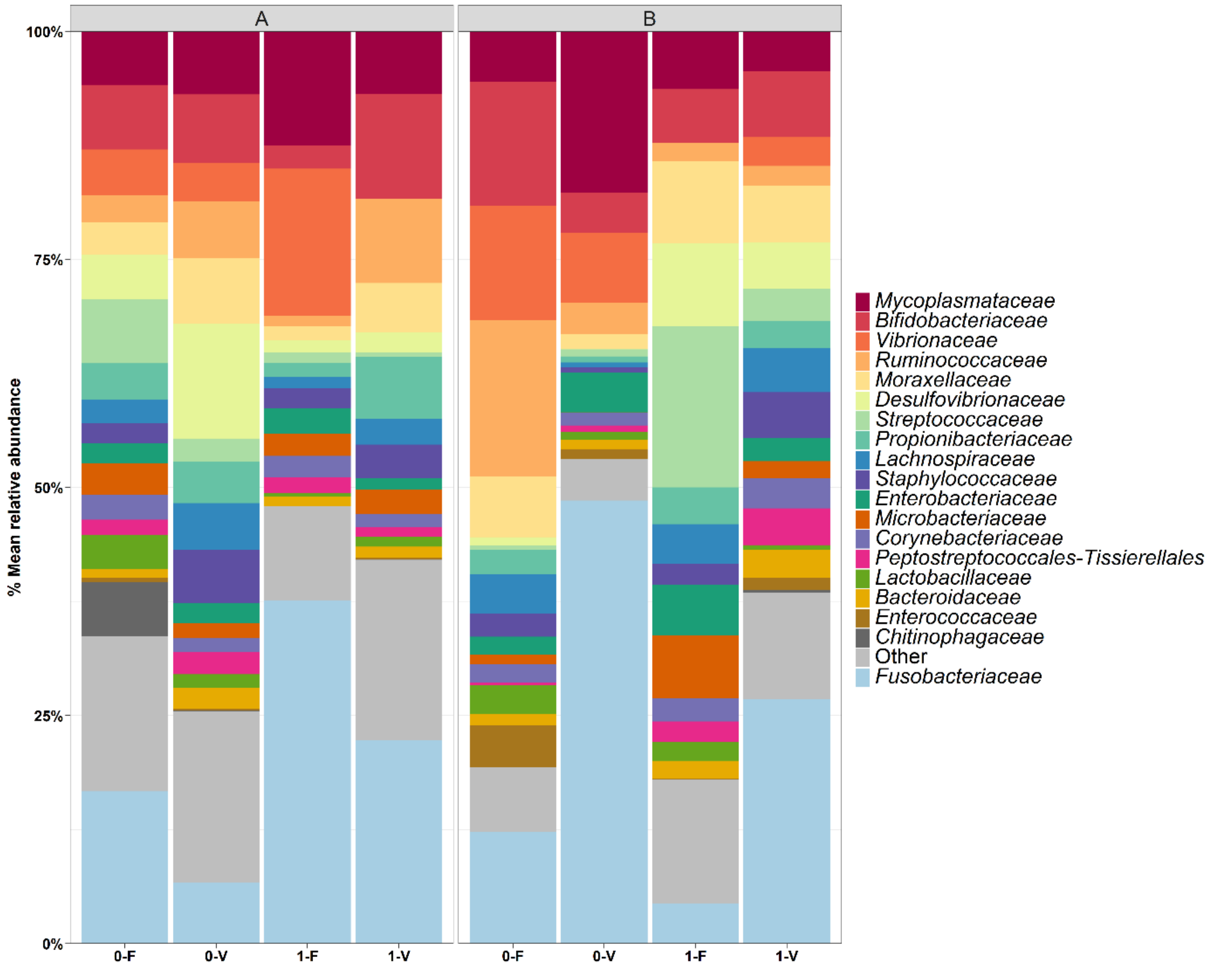

Figure S5 *Mean relative abundance (%) at the family level of the intestinal microbiota from trout line A in left and B in the right panel. Each bar represents a diet * stress combination treatment within the trout genetic lines. Genera with a representation of 1.5 % were pooled in category 'Other'. The arrangement of the bars is based on abundance, except for the most abundant phyla, which is placed at the bottom for legibility. The mean value joins data on each individual fish for the corresponding treatment across three modules.*
